# Global identification and characterization of miRNA family members responsive to potassium deprivation in wheat (*Triticum aestivum* L.)

**DOI:** 10.1038/s41598-020-72642-y

**Published:** 2020-09-25

**Authors:** Yong Zhao, Ke Xu, Gaoran Liu, Shanshan Li, Sihang Zhao, Xiaowei Liu, Xueju Yang, Kai Xiao

**Affiliations:** 1 State Key Laboratory of North China Crop Improvement and Regulation, College of Agronomy, Hebei Agricultural University, Baoding, 071000 Hebei China; 2 College of Life Sciences, Hebei Agricultural University, Baoding, 071000 Hebei China; 3 College of Resources and Environment Science, Hebei Agricultural University, Baoding, 071000 Hebei China

**Keywords:** Molecular biology, Plant sciences

## Abstract

Potassium (K) is essential for plant growth and stress responses. MicroRNAs (miRNAs) are involved in adaptation to nutrient deprivation through modulating gene expression. Here, we identified the miRNAs responsive to K deficiency in *Triticum aestivum* based on high-throughput small RNA sequencing analyses. Eighty-nine miRNAs, including 68 previously reported ones and 21 novel ones, displayed differential expression under K deficiency. In Gene Ontology and Kyoto Encyclopedia and Genome analyses, the putative target genes of the differentially expressed miRNAs were categorized into functional groups associated with ADP-binding activity, secondary metabolic pathways, and biosynthesis and metabolism. Functional characterization of tae-miR408, an miRNA significantly down-regulated under K deficiency, revealed its important role in mediating low-K tolerance. Compared with wild type, transgenic tobacco lines overexpressing tae-miR408 showed significantly improved K uptake, biomass, photosynthesis, and reactive oxygen species scavenging under K deficiency. These results show that distinct miRNAs function in the plant response to K deficiency through regulating target genes involved in energy metabolism and various secondary metabolic pathways. Our findings shed light on the plant response to K deficiency mediated by miRNAs in *T. aestivum*. Distinct miRNAs, such as tae-miR408, are valuable targets for generating crop varieties with improved K-use efficiency.

## Introduction

Potassium (K) is a critical inorganic nutrient that is essential for plant growth, development, and yield formation in cereal crops^1^. Many agricultural soils around the world are K deficient, which limits sustainable crop development^2^. In northern China, more than a quarter of the arable soils have an insufficient supply of available K. Therefore, K fertilizers are applied to increase crop productivity in intensive cropping systems^3^. However, frequent overuse of K fertilizers has caused serious environmental problems, as well as increasing farming costs^4^. Understanding the molecular mechanisms underlying plant responses to K deficiency will be useful for genetic improvement to increase low-K tolerance in various crops.

Members of the microRNA (miRNA) family are single-stranded RNA molecules, and their mature sequences are 21–23 nt in length. Previous studies have demonstrated that miRNAs play roles in regulating the plant response to abiotic stresses, such as inorganic nutrient deficits^5^. An miRNA up-regulated under K deficiency, miR156, was first identified in *Arabidopsis*. Later, other miRNAs such as miR169, miR395, and miR398, were also found to respond to K deficiency^6^. Functional analyses have shown that distinct miRNAs regulate plant adaptation to nutrient deficit. For example, expression of miR399 in *Arabidopsis* and rice improved tolerance to low-K and low-phosphorus (P) conditions via regulating target genes involved in nutrient acquisition and internal nutrient translocation^7^. *Arabidopsis* miR444a was found to be up-regulated under both low-K and low-N conditions. Its target genes, *MADS-23*, *MADS-27a*, *MADS-27b*, and *MADS-57*, encode transcription factors in the MAD family, which are post-transcriptionally regulated and endow plants with enhanced tolerance to nutrient deprivation^8^. Other studies have shown that the module miR319/TCP4 and the pathways mediated by the module miR396/GRF contribute to high tolerance to low-K stress in barley, and that ata-miR1432-5p acts as a regulator in Ca^2+^ signaling transduction pathways initiated by low-K stress^9^. In addition, stu-miR530_L-2R+2 in potato mediates responses to K treatments via regulation of a gene encoding a Zinc Knuckle (CCHC-type) family protein^10^. Together, these findings indicate that miRNAs are involved in mediating plant responses to nutrient stress.

As one of the highly conserved miRNA family members across diverse plant species^11^, miR408 plays critical roles in coordinating the balance between plant growth and responses to various stresses^12–14^. In a previous study, overexpression of miR408 in *Arabidopsis* conferred plants with enhanced tolerance to low temperature, oxidative stress, and salinity, but increased their sensitivity to drought stress^15^. Several reports have focused on the functions of miRNAs in mediating nutrient metabolic pathways in various plant species. For example, miR408c in soybean is highly induced under Pi-depleted conditions^16^; miR408 in barley mediates the response to low-K via regulation of its target gene encoding blue copper protein (BCP)^9^; and miR408 in cotton post-transcriptionally regulates its target gene encoding Cu/Zn superoxide dismutase 1A under nitrogen deficiency, to improve scavenging of reactive oxygen species (ROS)^17^. These findings suggest that miR408 acts as a crucial regulator and is involved in multiple biological processes.

Previous studies to identify members of the miRNA family, characterize their functions in mediating stress responses, and predict their target genes have been extensively performed in model plants, such as *Arabidopsis* and rice. Few studies have tried to identify miRNA members and characterize their functions in the K deficiency response in *Triticum aestivum*. In this study, we systematically identified the members of the miRNA family induced in *T. aestivum* under K deficiency through high-throughput small RNA sequencing analyses. The putative target genes of the miRNAs induced under K deficiency were identified and subjected to Gene Ontology (GO) and Kyoto Encyclopedia of Genes and Genomes (KEGG) analyses. We also functionally characterized tae-miR408, an miRNA showing drastic down-regulation under K deficiency, in terms of its roles in tolerance to K deficiency. Our results provide novel insights into the miRNA-mediated adaptation to low-K stress in *T. aestivum*.

## Results

### Quality of miRNA data

The miRNA sequencing data exhibited low base error rates (< 0.01%) and high Q20 and Q30 values (97.97% and 95.27%, respectively) (Table S1), confirming that the data were reliable and credible. The numbers of clean reads derived from samples at the six time points were 11, 921, 119 (CK, 0 h), 13, 868, 652 (LK-6, 6 h of K deficiency), 12, 075, 851 (LK-12, 12 h of K deficiency), 13, 967, 418 (LK-24, 24 h of K deficiency), 12, 310, 801 (LK-48, 48 h of K deficiency) and 18, 666, 289 (LK-120, 120 h of K deficiency). These quantities were in the range required for high-quality miRNA libraries.

The lengths of the miRNA molecules detected in libraries were 20–24 nt in most cases, with the 24 nt and 21 nt miRNAs being the most abundant types (Figure S1). The miRNA molecules identified were consistent with those reported previously in other plant species. Among the clean reads, more than 75.85% generated from various libraries were mapped to the reference genome of *T. aestivum* (cv. Chinese Spring) (Table S2). The quality analyses confirmed that the constructed libraries were of high quality, and the identification of miRNA molecules was accurate.

### miRNAs identified in K-deprived root tissues

The clean reads in the constructed miRNA libraries were subjected to mapping analysis against the reference sequence using the online tool miRNABase (https://www.mirbase.org/). We detected 87 different miRNA molecules. The alignment results between these miRNA members and known miRNA molecules are shown in Table S3. Aside from the miRNAs already listed in the database, a large set of miRNAs in the libraries were newly identified (Table S3). The sequences, nucleic acid lengths, and occurrence frequencies of the known miRNAs members are summarized in Figure S2.

### Differentially expressed miRNAs under K deficiency

To analyze the expression patterns of the miRNAs identified at various time points under K deficiency, we quantified the expression levels of miRNAs in wheat roots under low-K conditions (6, 12, 24, 48, and 120 h) and in the control (0 h) (Supplementary Table S4). Based on the log2.Fold_change (FC) values in five comparisons (LK-6/CK, LK-12/CK, LK-24/CK, LK-48/CK, LK-120/CK), the up-regulated miRNA members with the highest expression levels were tae-miR395a (FC of 8.82 between LK-120 and CK) and the newly identified miRNA novel_50 (FC of 8.54 between LK-48 and CK). Likewise, the miRNAs with the most down-regulated expression were tae-miR9657a-3p (FC of − 9.19 between LK-6 and CK) and novel_51 (FC of − 7.97 between LK-6 and CK). In total, 51 miRNAs, including 37 known ones and 14 newly identified ones, were differentially expressed in LK-6 compared with CK (26 up-regulated and 25 down-regulated). Likewise, 47 miRNAs, including 33 known ones and 14 novel ones, were differentially expressed in LK-12 compared with CK (25 up-regulated and 22 down-regulated). Forty-six miRNAs, including 32 known ones and 14 newly identified ones, were differentially expressed in LK-24 compared with CK (27 up-regulated and 19 down-regulated). Thirty-five miRNAs including 26 known ones and nine newly identified ones were differentially expressed in LK-48 compared with CK (20 up-regulated and 15 down-regulated). Forty-five miRNAs including 37 known ones and eight novel ones were differentially expressed in LK-120 compared with CK (26 up-regulated and 19 down-regulated).

Among the differentially expressed miRNAs mentioned above, miR9772, miR1120b-3p, miR531, and miR319 were differentially expressed at all time points during the low-K treatment (Figure S3). As shown in Table S5, five miRNAs (novel_50, miRNA531, miRNA9772, miRNA9670-3p, and miRNA9773) were up-regulated and seven (novel_17, miRNA408, miRNA1127a, miRNA159a, miRNA319, miRNA398, and miRNA9778) were down-regulated in more than three comparison groups with FC values ranging from − 6.22 to 8.53. For example, miR408, a down-regulated miRNA, had FC values of − 5.08, − 4.07, and − 5.52 FC in comparison groups LK-6/CK, LK-12/CK, and LK-24/CK, respectively. These results suggest that this miRNA is possibly involved in mediating plant adaptation to K deficiency.

### Prediction of target genes of differentially expressed miRNAs

We predicted the target genes of the following 12 differentially expressed miRNAs: novel_17, novel_50, miRNA319, miRNA531, miRNA9773, miRNA9670-3p, miRNA398, miRNA159a, miRNA9778, miRNA408, miRNA9776, and miRNA1133. The putative target genes of these miRNAs are shown in Supplementary Table S6. Genes encoding RING-type E3 ubiquitin transferase and glycosyltransferase were among the predicted target genes of the newly identified miRNA novel_17. These enzymes are involved in regulating internal K metabolism. The target genes of known miRNAs included those encoding the RNA-dependent RNA polymerase and peroxidase (targeted by miR159a), auxin-responsive protein and peroxidase (targeted by miR319), superoxide dismutase [Cu–Zn] and chloride channel protein (targeted by miR398), NAC domain protein and protein phosphatase (targeted by miR408), alpha-mannosidase I and metal-nicotine transporter (targeted by miR531), transposons TNT 1-94 and formalin 6 (targeted by miR9773), serine/threonine protein kinase (targeted by miR9670-3p and miR11330, and lipoxygenase (targeted by miRNA9776) (Table S6). These results strongly suggest that complex biological networks are involved in the low-K response of wheat.

### Functional classification of target genes

**Figure 1:**
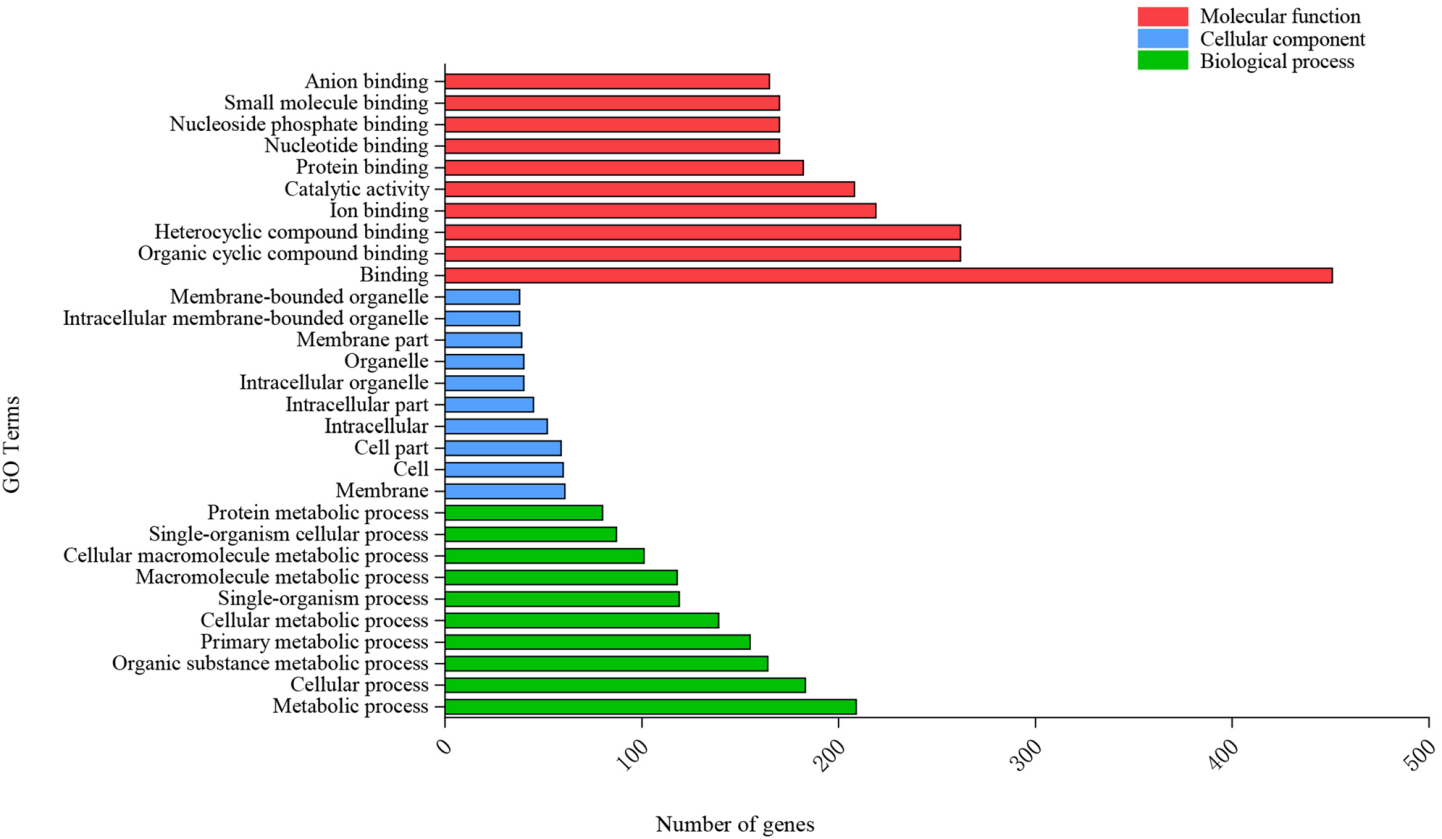
GO enrichment results for target genes of miRNAs.

To understand the functions of the genes targeted by miRNAs showing differential expression under K deficiency, the target genes of the differentially expressed miRNAs mentioned above were subjected to GO enrichment analysis. A total of 985 GO terms were identified: 579 in the biological processes category, 282 in the molecular function category, and 124 in the cellular components category. The top ten GO terms in each of the three major categories are shown in Fig. 1. Binding, membrane, and metabolic process were the most enriched terms in the molecular function, cellular component, and biological process categories, respectively. The GO terms included genes encoding potassium ion transmembrane transporters, a protein involved in superoxide metabolism (encoded by a superoxide dismutase [Cu–Zn] gene targeted by miR398), and proteins involved in ADP-binding and carbohydrate derivative binding. These gene products are possibly involved in the response to K deficiency. These annotation results are valuable for identifying the biochemical pathways involved in the plant response to K deficiency.

**Figure 2:**
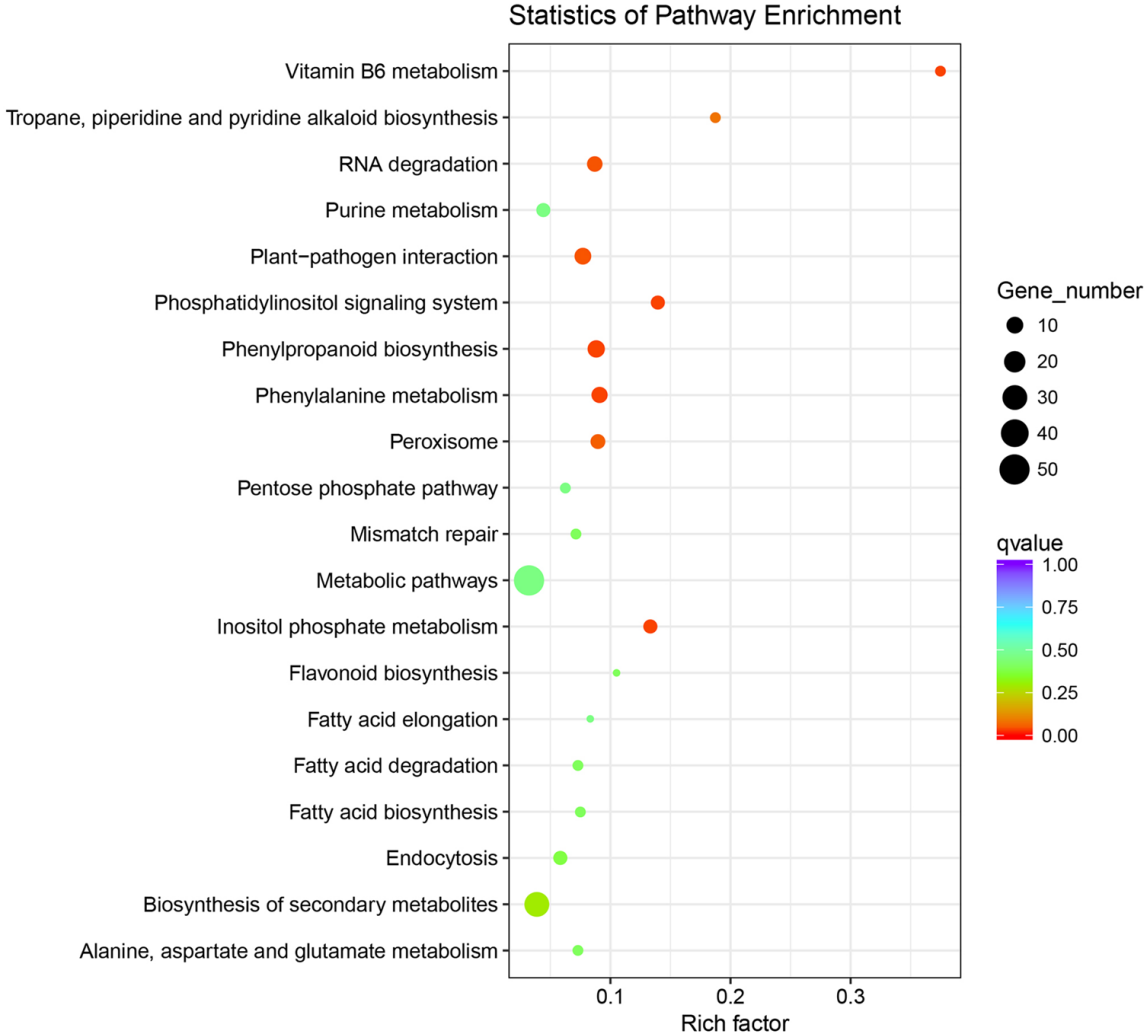
KEGG metabolic processes enriched with miRNA target genes.

Next, a KEGG enrichment analysis of the target genes revealed that they were associated with photosynthesis, potassium uptake, and secondary metabolite biosynthesis. The products of the target genes were also involved in biochemical processes related to RNA degradation, plant-pathogen interactions, the phosphatidylinositol signaling system, phenylpropanoid biosynthesis, phenylalanine metabolism, and peroxisome biological pathways (Fig. 2). For example, the differentially expressed gene *POD* targeted by miR319 encodes a protein involved in biosynthesis of secondary metabolites, metabolic pathways, phenylpropanoid biosynthesis, and phenylalanine metabolism. Thus, biochemical processes, especially those associated with carbon assimilation and nutrient transport, are involved in the K-deprivation response of *T. aestivum*.

### Validation of transcript abundance of target genes

**Figure 3:**
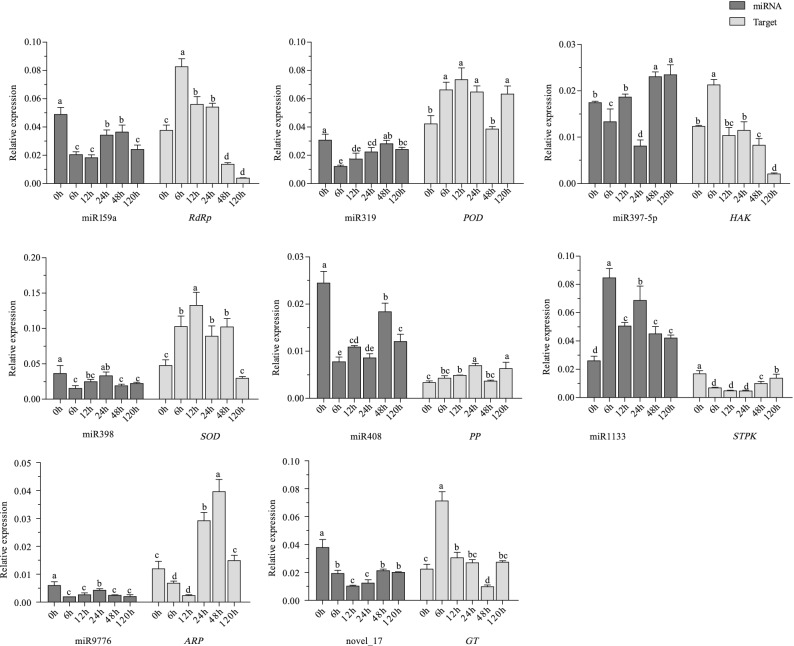
Expression patterns of eight selected miRNAs and their target genes in roots of wheat cultivar Kenong 9204 after 0, 6, 12, 24, 48, and 120 h of low-K treatment. Different letters above columns indicate significant differences at *p* < 0.05 (Duncan’s multiple range test). *RdRP*, a target of miR159a encoding RNA-dependent RNA polymerase; *POD*, a target of miR319 encoding peroxidase; *HAK*, a target of miR397-5p encoding potassium transporter; *SOD*, a target of miR398 encoding superoxide dismutase [Cu–Zn]; *PP*, a target of miR408 encoding a protein phosphatase; *STPK*, a target of miR1133 encoding serine/threonine-protein kinase; *ARP*, a target of miR9776 encoding auxin-responsive protein; *GT*, a target of novel_17 encoding glycosyltransferase.

Eight of the miRNAs identified as being differentially expressed under K deficiency in analyses of miRNA libraries were analyzed by qRT-PCR to validate their expression patterns. These eight miRNAs were miR9776, miR398, miR397-5p, miR319, miR1133, miR159a, miR408, and novel_17. The results indicated that expression levels of the miRNA members examined were all comparable with the miRNA sequencing results. We also analyzed the expression patterns of the following genes targeted by the miRNAs: *ARP*, *SOD*, *HAK*, *POD*, *STPK*, *RdRp*, *PP* and *GT* (the target genes of miR9776, miR398, miR397-5p, miR319, miR1133, miR159a, miR408, and novel_17, respectively). These genes showed the opposite expression patterns to those of their targeting miRNAs (Fig. 3). For example, miR408 was down-regulated under K-starvation conditions (FC − 2.85) while its target gene was up-regulated (FC 2.08 at 24 h compared with 0 h). Comparing relative expression levels at 6 h and 0 h, miR1133 was up-regulated (FC 3.26) and its target was down-regulated (FC − 2.46). The expression patterns of the target genes were negatively correlated with those of their targeting miRNAs. These results suggest that the target genes are negatively regulated by miRNAs via a posttranscriptional mechanism.

### Role of tae-miR408 in mediating K-starvation tolerance

**Figure 4:**
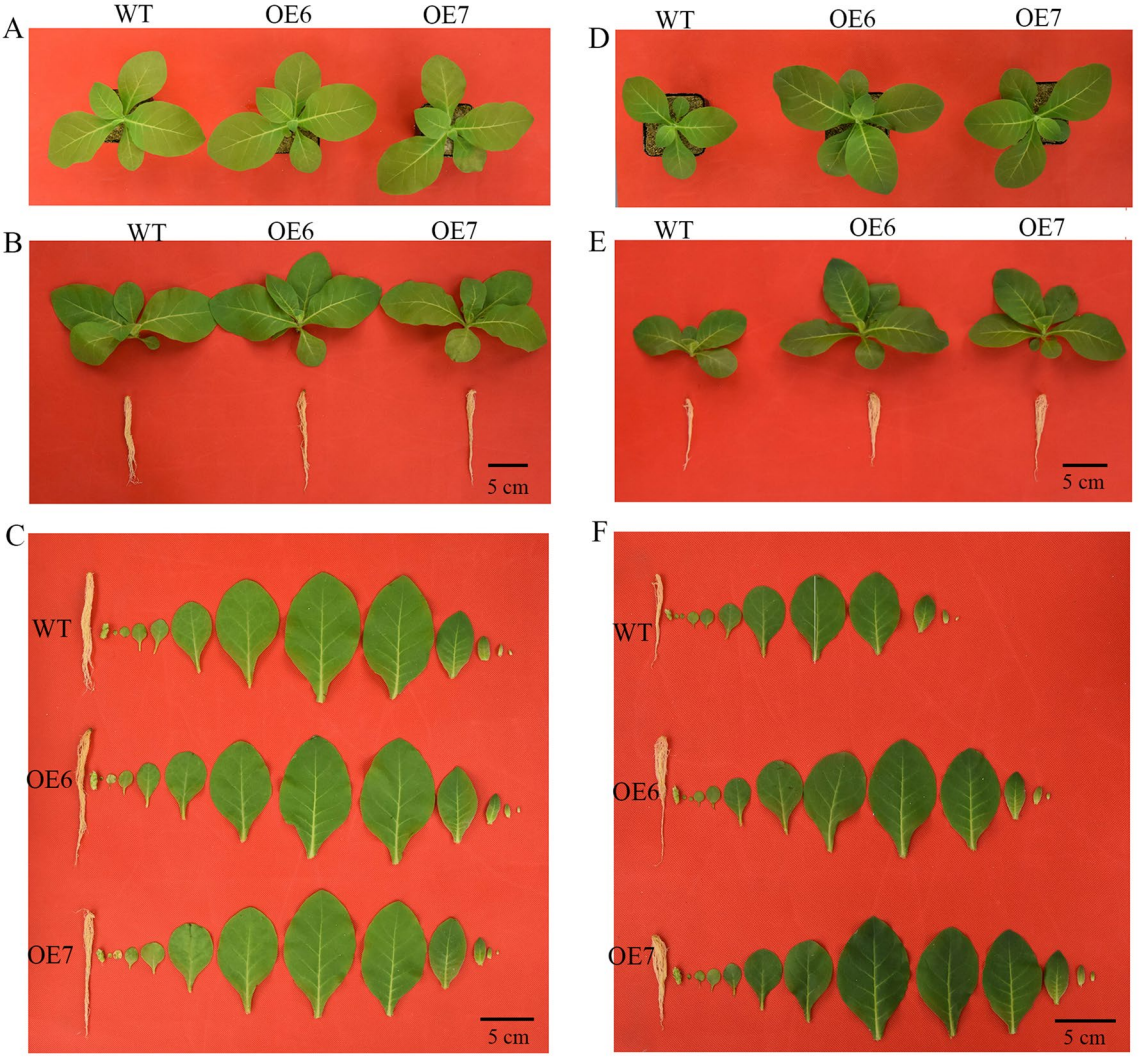
Phenotypes of *Nicotiana tabacum* lines overexpressing tae-miR408 under control (MS) and low-potassium (LK) conditions. OE6 and OE7, two tobacco lines overexpressing tae-miR408; WT, wild type. (**A**–**C**), WT, OE6, and OE7 lines under MS; (**D**–**F**) WT, OE6, and OE7 lines under LK. Scale label in (**B**, **C**, **E**, **F**) = 5 cm.

**Figure 5:**
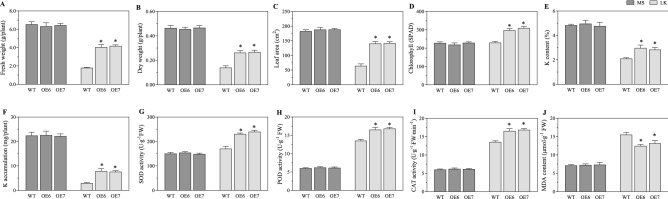
Tae-miR408 overexpression confers low potassium (K) stress tolerance in transgenic *Nicotiana tabacum.* MS, control; LK, low-potassium; OE6 and OE7, two tobacco lines overexpressing tae-miR408; WT, wild type. (**A**) fresh weights; (**B**) dry weights; (**C**) leaf areas; (**D**) chlorophyll content; (**E**) K content; (**F**) K accumulation; (**G**) superoxide dismutase activity; (**H**) peroxidase activity; (**I**) catalase activity; (**J**) malondialdehyde content. Data are averages of triplicate experiments plus standard errors; * indicates significant differences at *p* < 0.05 (Duncan’s multiple range test).

Our results show that tae-miR408 was the most strongly down-regulated miRNA, and showed differential expression at more than three treatment time points under K deficiency (Supplementary Table S5). Therefore, this miRNA was selected for functional analyses. Under sufficient-K conditions, the wild type (WT) and OE6 and OE7, two T3 lines overexpressing tae-miR408, had comparable phenotypes (Fig. 4) in terms of fresh weight, biomass, leaf area, chlorophyll and K contents, activities of superoxide dismutase (SOD), peroxidase (POD), and catalase (CAT), and malondialdehyde (MDA) content (Fig. 5A–J). Under K-deficient conditions, however, OE6 and OE7 showed significantly improved phenotypes (leaf area, fresh weight, and biomass), physiological and biochemical parameters (chlorophyll content, K content, activities of CAT, SOD, and POD), and lower MDA contents, compared with WT (Fig. 5A–J). The fresh weight (Fig. 5A) and leaf area (Fig. 5C) in OE lines were more than double those in WT under K-deprivation conditions, but were almost comparable under control conditions. Under normal conditions, the OE lines and WT accumulated similar amounts of K (22.21, 22.45, and 22.59 mg per plant in WT, OE6, and OE7, respectively). Under K-deficient conditions, however, the OE lines accumulated more K than did WT (2.94, 7.81 and 7.59 mg per plant in WT, OE6, and OE7, respectively) (Fig. 5F). These results indicate that miR408 endowed plants with enhanced K accumulation capacity under low-K conditions, a trait that is essential for plants to adapt to K deficiency.

**Figure 6:**
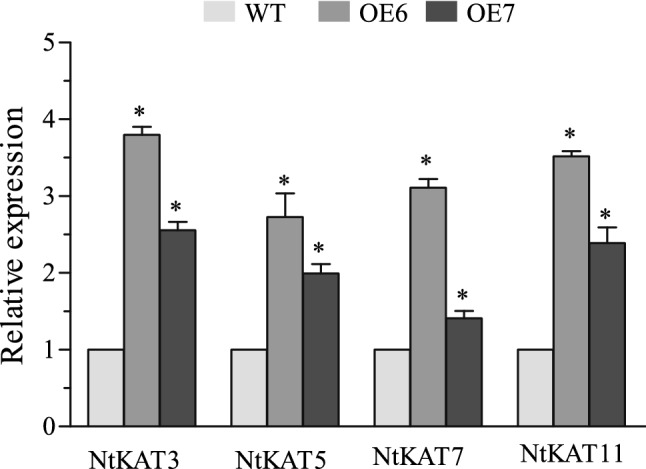
Transcript levels of *KAT* detected by qRT-PCR in transgenic tobacco lines overexpressing tae-miR408 after 24 h of low-potassium treatment. WT, wild type; OE6 and OE7, two tobacco lines overexpressing tae-miR408. Data are averages of triplicate experiments plus standard errors; *indicates significant differences at *p* < 0.05 (Duncan’s multiple range test).

The expression patterns of a set genes encoding K ion channels (KAT) were analyzed in the OE lines under K-deprivation conditions, to determine their contribution to K deficiency adaptation in the tae-miR408-overexpressing lines. *NtKAT3*, *NtKAT5*, *NtKAT7,* and *NtKAT11* were drastically up-regulated in the OE lines compared with WT, whereas other members of the KAT gene family showed no differences in transcript levels between OE and WT plants (Fig. 6). *TaAKT1*, which encodes an AKT1-like potassium channel in *T. aestivum* and shares high homology with the *N. tabacum* K channel gene mentioned above, was also significantly up-regulated under K deficiency in wheat (Figure S4). These results suggest that these KAT genes regulated by tae-miR408 positively affect K uptake and/or internal K translocation in the tae-miR408-overexpressing lines with enhanced tolerance to K-deficient conditions.

## Discussion

**Figure 7:**
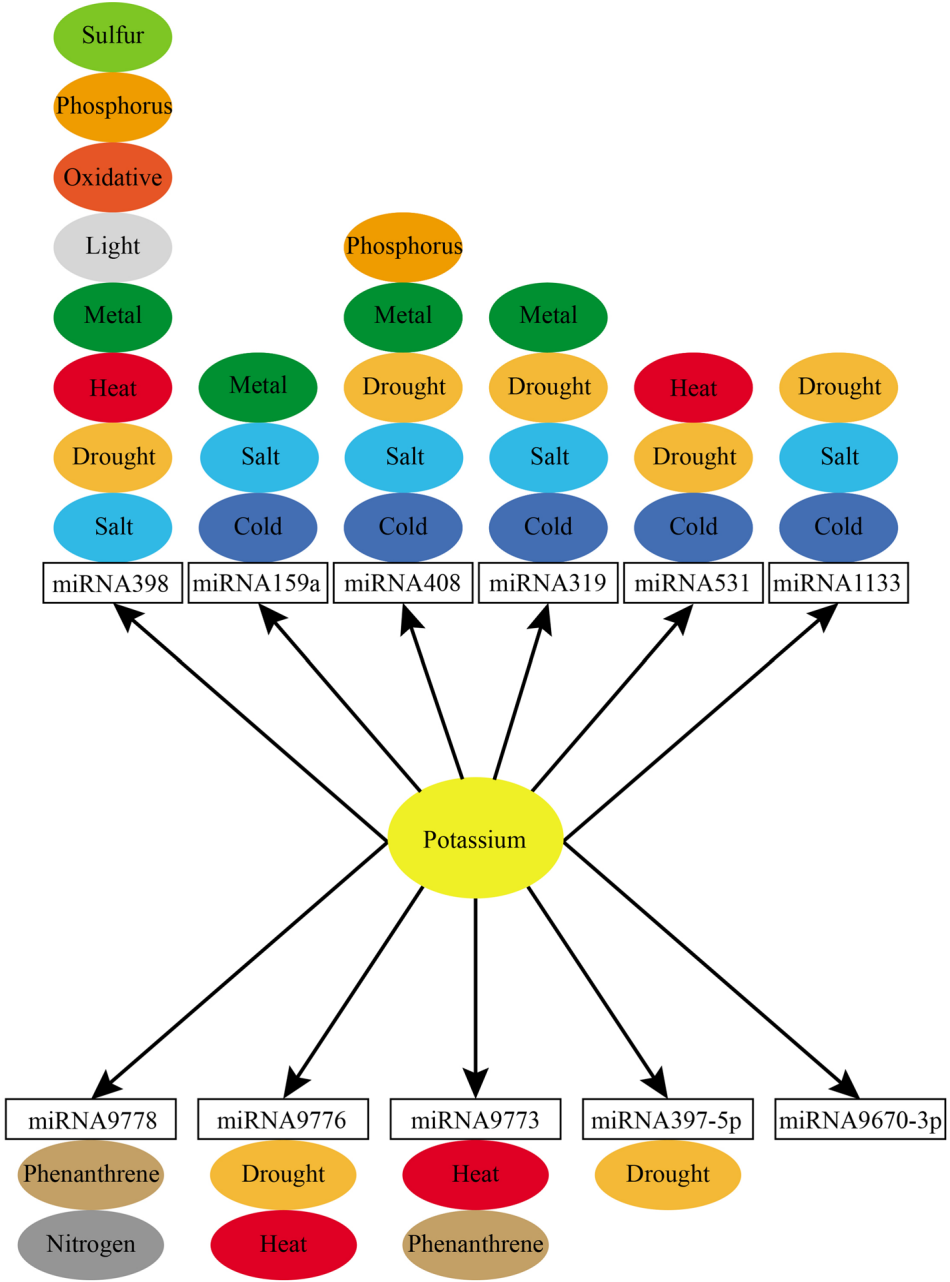
Functional map inferred from differentially expressed microRNAs under low-potassium conditions.

Plants have evolved sophisticated mechanisms to cope with environmental stresses, and these mechanisms are closely associated with distinct miRNA members (Fig. 7)^18–22^. Members of the miRNA family are involved in the regulation of various biological processes in plants through modulating target genes at posttranscriptional and/or translational levels^23–25^. For instance, miR9773, miR531, and miR9778 are responses to phenanthrene (an organic pollutant) by regulating phenanthrene metabolism^26^. miR319 and miR395 regulate plant adaptation to nitrogen deprivation through modulating transcription of the genes involved in nutrient metabolism^27^. Additionally, miR319 has been recorded to be also involved in mediating plant response to Pi starvation stress^28^, endowing improved plant growth and stress tolerance by regulating chitosan metabolism^29^ and inhibiting viroid infection into spindle tuber^30^. miR398 is down-regulated upon oxidative stress^31^, playing an important role in modulating the regulatory networks associated with ROS scavenging, water deficit, salt stress, UV stress, abscisic acid stress, and Cu and Pi deprivation^32,33^. The SOD encoding gene targeted by miR398 contributes to the stabilization of antioxidant enzymes and heat shock proteins^34^, by which to confer plant responses to high Cu^2+^^35–37^, drought stress^38^, ozone, and salinity^39^, avirulent *Pseudomonas syringae* infection^40^, SO_2_-induced oxidative stress^41^, and phenanthrene stress^26^. The members in the miR408 family acts as a crucial regulator in plant responses to cold, salt, drought, Pi and metal^15,16^. In this study, based on high-throughput miRNA sequencing analysis, we systematically investigated members of the miRNA family induced in *T. aestivum* under K deficiency. Our results revealed that a large set (in total of 89) miRNA family members were significantly modified in expression in root tissues upon K starvation condition, with either up-regulated pattern or down-regulated one. It is noted to mention that one of them referred to as tae-miR408 was shown to be significantly expressed in a suite of time points checked under K deprivation condition, suggesting its putative roles in the mediation of plant K deficiency adaptation.

The target genes of miRNAs function in various biological processes, such as transcriptional regulation^42^, primary metabolism^43^, secondary metabolism^44^, phytohormone responses^45^, and ROS scavenging^39,46^. In this study, we predicted the target genes of the differentially expressed miRNAs under K deficiency, and then conducted GO and KEGG analyses on them to explore the putative target biological roles. Our functional characterization on the target genes revealed that they are extensively involved in association with plant growth, physiological and biochemical processes, and abiotic stress responses. For example, the auxin-responsive gene targeted by miR319 plays critical roles in regulating growth and development in *Arabidopsis*^47^; the target gene of miR159a encodes an RNA-dependent RNA polymerase and involves development processes such as floret initiation and flowering progression^48,49^; and the gene targeted by miR9773 that codes for retrovirus-like transposon Tnt1-94 modulates various physiological processes associated with tissue establishment^50^. Additionally, a set of the target genes underlying regulation of the differentially expressed miRNAs have also been confirmed to be involved in plant stress adaptation. For example, a target gene of miR398 that encodes a SOD [Cu/Zn] mediates cellular copper homeostasis upon Cu deprivation^51,52^. The gene regulated by miR9776 that codes for a lipoxygenase plays essential roles in lipid metabolism and confers plant osmotic stress response^53^. The target gene of miR408 encodes a phosphatase, which promotes cellular Cu translocation across tissues under drought conditions^54,55^. In addition, two target genes of miR9778 that are associated with anti-pathogen infection processes are involved in the plant pathogen response through alleviation of pathogen infection^56^. In this study, we systematically identified the putative target genes interacted with the differential miRNAs. Our results suggested that the target genes were specifically enriched in the functional categories associated with ADP-binding activity and secondary metabolic pathways, suggesting that the miRNA-mediated K-deficiency response largely ascribes to functions of the miRNA members in regulating the energy and secondary metabolisms. Altogether, these results indicate the critical roles of distinct miRNAs in plant responses and acclimation to diverse stresses, such as potassium deficiency, through the cross-talk among diverse physiological processes and biochemical pathways underlying distinct miRNA/target modules. Further characterization of the miRNAs mentioned above can reveal commonalities among the miRNA-mediated K-deprivation responses in various plant species.

miR408 regulates both growth and stress responses in plant species. For example, transgenic *Arabidopsis* lines overexpressing miR408 showed altered morphology and significantly increased leaf area, petiole length, plant height, flower size, pod length, and seed yields^57^. In rice, posttranscriptional modification of the target *OsUCL8* by miR408 improves cellular Cu homeostasis, plastocyanin function, photosynthesis, and yield formation capacity^57^. Expression of miR408 in chickpea plants enhanced their drought tolerance, improved their phenotype, and increased plant biomass under water deficit^38^. In wheat, miR408 is induced under Pi starvation, and mediates the responses to P starvation and drought by regulating the transcription of *TaTOC1*^58^. These reports illustrate the diverse roles of miR408 in mediating various stress responses. In this study, our expression analysis on members of the miRNA families revealed that tae-miR408 was significantly down-regulated under K deficiency, whose transcripts were shown to be drastically lowered at various indicated tome points under K-depleted treatment with respect to control (prior to K deprivation treatment). These findings prompted us to characterize its function in mediating tolerance to K deficiency in more detail, based on transgene analysis in an ectopic expression system (*N. tabacum*). Results indicated that the transgenic lines with miR408 overexpression showed drastic improvement on phenotypes, biomass, protective enzyme activities, and chlorophyll contents compared with wild type under K deficiency. These results confirm that tae-miR408 is critical in mediating plant K deficiency tolerance and is a valuable target in generating new crop cultivars with high K-use efficiency.

In plants, K regulates the growth and development as well as stress responses of plants due to the role in modulating diverse biochemical and physiological processes, such as osmolyte metabolism and cellular water homeostasis under abiotic stress^59–61^. The absorption and transport of K are mediated by K ion channels, therefore, these proteins greatly contribute to K nutrition in plants^62,63^ and stomatal movement in guard cells^64^. To determine whether any KAT family genes were involved in regulating K uptake in the transgenic lines, we analyzed the expression patterns of a set of KAT genes in the transgenic lines under K deficiency. We found that the transcripts of four KAT genes, including *NtKAT3*, *NtKAT5*, *NtKAT7*, and *NtKAT11*, were significantly up-regulated in the transgenic lines compared with WT. Recently, Huang et al.^65^ reported the up-regulated expression of *NtKAT3* in *N. tabacum* under low-K conditions, suggesting that it is involved in plant K-deficiency response. This finding, together with the K ion channel gene expression patterns detected in this study, suggest that the improved tolerance to K deficiency conferred by tae-miR408 is at least partly due to the up-regulated expression of some KAT genes. Further research is required to explore the role of tae-miR408 in regulating the transcription of K channel genes under stress conditions.

Under K deficiency, ROS accumulate more in cells^66^ that can damage cellular components and negatively affect various biological processes^67^. Shin et al.^68^ reported that the ROS induced in *Arabidopsis* roots under K deficiency also act as the signaling molecules in the K-starvation signaling pathway. On the other hand, plants scavenge excess ROS with a suite of antioxidant enzymes, such as POD, SOD, and CAT^69,70^, which alleviate the toxicity of ROS, thereby preventing lipid peroxidation and protecting cellular function under stress conditions^71^. In this study, we found that the cellular ROS homeostasis-associated parameters, such as the activities of CAT, SOD, and POD, as well as MDA contents, were drastically modified by tae-miR408. Compared with WT, OE6 and OE7 displayed higher activities of POD, SOD, and CAT and lower contents of MDA under K deficiency. Thus, the transgenic lines that showed enhanced tolerance to K deficiency was associated with the improved cellular ROS homeostasis, which positively affects K uptake and plant growth under K-deficient conditions.

## Conclusions

miRNA sequencing analysis identified in total of 89 miRNA family members in *T. aestivum* that are differentially expressed under K deficiency condition, including 68 of known and 21 of novel identified ones. The target genes of 11 differentially expressed miRNAs (novel_17, miRNA319, miRNA531, miRNA9773, miRNA9670-3p, miRNA398, miRNA159a, miRNA9778, miRNA408, miRNA9776, and miRNA1133) are associated with the plant tolerance to K deficiency in *T. aestivum*. GO and KEGG analysis revealed that the target genes of differentially expressed miRNAs were especially enriched in functional categories associated with ADP-binding activity and secondary metabolic pathways, suggesting that the essential roles of miRNAs are concentrated on energy and secondary metabolism during the plant K-deficiency response. miR408 transcripts are significantly down-regulated under low-K conditions, and its target gene is up-regulated. Overexpression of tae-miR408 confers plants improved morphology and enhanced K-deficiency tolerance through improvement of K uptake, photosynthetic pigment biosynthesis, and ROS homeostasis. Our study provides novel insights in the miRNA-mediated response to K deficiency in *T. aestivum*.

## Materials and methods

### Plant materials

We used Kenong 9204, a wheat cultivar released by the Chinese Academy of Sciences, China^72^. This cultivar was shown to be tolerant to K deficiency (unpublished data, Figures S5 and S6).

### K-deficiency treatment

Seeds (cv. Kenong 9204) were germinated after disinfection with 75% alcohol and washing with deionized water. Uniform seedlings were then grown in standard MS solution (6 mmol L^−1^ K) for 12 days under the following growth conditions: relative humidity of 70%, photoperiod of 12 h/12 h (day/night), at 25 °C/23 °C (light/dark). The seedlings were then transferred into a modified MS solution containing only 15 µmol L^−1^ K to impose K deficiency. At 0, 6, 12, 24, 48 and 120 h of this treatment, root tissues from five individual plants were sampled, frozen immediately in liquid nitrogen, and stored at − 80 °C until high throughput sequencing and other analyses.

### RNA extraction

Total RNA was extracted from roots sampled at various times using the TRIzol reagent kit according to the manufacturer’s instructions. The quality and integrity of RNA were evaluated based on agarose gel electrophoresis and the OD260/280 ratio detected using a Nanodrop spectrophotometer (Nanodrop Technologies, Wilmington, DE, USA). RNA degradation and contamination were monitored by electrophoresis on 1% agarose gels. RNA purity was checked using the Nano Photometer spectrophotometer (Implen, Munich, Germany). The RNA concentration was measured using Qubit RNA Assay Kit with a Qubit 2.0 Flurometer (Life Technologies, Carlsbad, CA, USA). RNA integrity was assessed using the RNA Nano 6000 Assay Kit and the Agilent Bioanalyzer 2100 system (Agilent Technologies, Santa Clara, CA, USA).

### Construction of miRNA libraries

The miRNA libraries for root tissues were constructed and sequenced using the Illumina Hiseq-2000 platform. Briefly, 3 μg total RNA per sample was used to construct miRNA libraries using the NEB Next Multiplex Small RNA Library Prep Set for Illumina (NEB, Ipswich, MA, USA). Using T4 RNA ligase, the miRNA was ligated with 3′- and 5′-end adapters, then first-stand cDNA was synthesized using M-MuLV Reverse Transcriptase (RNase H^-^). The synthesized cDNA was further subjected to PCR amplification. The DNA was purified by electrophoresis on a 8% polyacrylamide gel at 100 V for 80 min. The amplified DNA fragments (140–160 bp) were used to construct miRNA libraries. Library quality was assessed using the DNA High Sensitivity Chips of the Agilent Bioanalyzer.

### Bioinformatics analysis of sRNA sequencing data

The miRNA reads in constructed libraries were generated by Illumina HiSeq™ analysis. Briefly, the raw sequencing data from the libraries were pre-processed to remove low-quality reads (reads shorter than 18 nt in length, adaptor sequences, and false small molecules formed via adaptor-adaptor ligation). The GC-content, Q20, and Q30 values were calculated. The sRNA molecules were then subjected to mapping analysis against the genome reference sequence of *T. aestivum* using Bowtie software. Based on molecule structure, the miRNA reads were categorized into the following groups: exon, intron, rRNA, tRNA, snRNA, snoRNA, repeat-associated RNA, ta-miRNA, and ntRNA. Furthermore, the miRNA members were identified by BLAST algorithm-based searches against the miRBase 21 database (https://www.mirbase.org/). miRNAEvo^73^ and miRNAdeep2^74^ were used to predict the novel miRNA members.

### Identification of differential miRNA family members

TPM analysis was performed to identify the differentially expressed miRNA members in the miRNA libraries^75,76^. The differential miRNA members were defined as those with a q value of < 0.01 and the |log2(fold change)| of > 1. The target genes of known and newly identified miRNA members were predicted using the online tool psRNATarget (https://plantgrn.noble.org/psRNATarget/) following the suggested procedure. Then, a GO analysis was carried out to define the functional categories of the target genes. The GO term for a candidate target gene was determined based on its closeness to a certain category. Biochemical pathways that involve the candidate target genes were identified in a KEGG analysis^77,78^, where the targets were used as queries to scan against the entire *T. aestivum* genome database.

### Quantification of expression levels of miRNAs and their target genes

The cDNAs derived from roots at various time points under low-K conditions (0, 6, 12, 24, 48, and 120 h) were subjected to qRT-PCR analysis to validate the miRNA sequencing results. The genes analyzed by qPCR and the gene-specific primers used are shown in Table S7. *GAPDH*, a constitutive gene in *T. aestivum* species, was used as the internal reference to normalize target transcripts. qRT-PCR was performed on Light Cycler480 (Roche, Switzerland) using reagent Light Cycler 480 SYBR Green I (Roche) under the following thermal cycling program: 95 °C for 5 min, 40 cycles of 95 °C for 10 s and 60 °C for 30 s, followed by a denaturing step to generate the melting curve. All of the reactions were performed in biological triplicate, and produced comparable results. The gene transcript levels were calculated using the 2^−ΔΔCt^ method as previously reported^79^.

### Functional analysis of Tae-miR408 in mediating tolerance to K deficiency

Tae-miR408, an miRNA with down-regulated expression under K deficiency, was subjected to functional analysis to determine its role in mediating K-deficiency tolerance. To this end, RT-PCR was performed to amplify the precursor sequence of tae-miR408 using specific primers. The product was then inserted between the restriction sites *Nco*I/*BstE*II in the binary vector pCAMBIA3301 under the control of the constitutive CaMV35 promoter. The vector containing tae-miR408 was transformed into *Nicotiana tabacum* using an *Agrobacterium tumefaciens*-mediated approach. OE6 and OE7, two T3 lines with up-regulated tae-miR408 expression, were analyzed to determine their growth and physiological traits under K deficiency. Briefly, OE6 and OE7 and wild type (WT) were grown in plastic pots and supplied with standard MS solution under the following conditions: 28 °C/23 °C (light/dark), relative humidity of 70%, and a photoperiod of 12 h /12 h (light/dark) with light intensity of 300 µmolE m^−2 ^s^−1^ during the light phase. At the four-leaf stage, the transgenic and WT plants were subjected to normal K conditions (standard MS solution containing 6 mmol/L K) and K-deficient conditions (modified low-K MS solution containing 15 µmol/L K) for 21 d. After this treatment, the OE lines and WT were analyzed to determine their growth traits, K contents, and physiological parameters. Phenotypes were recorded using digital camera (Nikon D7200); leaf areas were measured using a portable leaf area analyzer; fresh weights were determined using an electronic balance; biomass was determined by weighing after oven drying the plant samples; K content was evaluated as described by Bao et al.^80^; activities of antioxidant enzymes including catalase (CAT), peroxidase (POD), and superoxide dismutase (SOD), together with malondialdehyde (MDA) contents, were determined using the methods of Li et al.^81^; and chlorophyll content was assessed using a chlorophyll meter (Konica Minolta SPAD-502 plus, Minolta Corp., Osaka, Japan). All of the physiological and biochemical parameters were measured in biological triplicate.

### Quantification of mRNAs of genes encoding K channels in wheat and tae-miR408-overexpressing lines

Several genes encode K channels that mediate K uptake in plants. The transcript levels of these genes were determined in the OE lines after a K-deficiency treatment to identify those involved in mediating tolerance to low-K conditions. To further understand the transcription of these gens in wheat plants under low-K stress, the transcript levels of *TaAKT1*, encoding an AKT1-like potassium channel in *T. aestivum* and sharing high homology with the *N. tabacum* K channel gene mentioned above, were assessed under low-K conditions. For this purpose, total RNA was extracted from OE6, OE7, and WT wheat plants under K-deficient conditions. Transcripts of the K channel genes were detected by qRT-PCR using the following procedure: 95 °C for 5 min, 40 cycles of 95 °C for 10 s and 60 °C for 30 s, followed by a thermal denaturing step to generate a melt curve. All reactions were performed in biological triplicate. Transcript levels were calculated from the qPCR data using the 2^−ΔΔCt^ method^79^. The genes examined and the specific primers used are shown in Supplementary Table S7.

### Statistical analyses

Average values of gene expression levels, growth traits, K contents, biomass, and chlorophyll contents were derived from biological triplicate results. Statistical Analysis System software (SAS Corporation, Cary, NC, USA) was used to test the significance of differences among mean values. Duncan’s multiple range test was used to identify significant (*p* < 0.05) differences among group averages based on analysis of variance (ANOVA).

## Supplementary information

Supplementary Information.

## Data Availability

All the information supporting the results of this manuscript was included in the article. GenBank accession number for the small RNA sequencing data defined in this paper is PRJNA523507.
