# Supplementary material for: Global identification and characterization of miRNA family members responsive to potassium deprivation in wheat (Triticum aestivum L.)
Source: Sci Rep. 2020 Sep 25;10:15812. doi: 10.1038/s41598-020-72642-y (PMC7519128; doi:10.1038/s41598-020-72642-y)
Supplement: Supplementary file 1 — Supplementary Information. [file 41598_2020_72642_MOESM1_ESM.pdf]

Supplementary Information

Global Identification and Characterization of miRNA Family Members Responsive to Potassium Deprivation in Wheat (*T. aestivum* L.)

Yong Zhao[1, a], Ke Xu[1, a], Gaoran Liu[2], Shanshan Li[1], Sihang Zhao[1], Xiaowei Liu[3], Xueju Yang[2], Kai Xiao[1,*]

[1] College of Agronomy, Hebei Agricultural University, Baoding 071000, Hebei, China.

[2] College of Life Sciences, Hebei Agricultural University, Baoding 071000, Hebei, China.

[3] College of Resources and Environment science, Hebei Agricultural University, Baoding 071000, Hebei,
China.

* Correspondence: xiaokai@hebau.edu.cn

[a] These authors contributed equally to this work

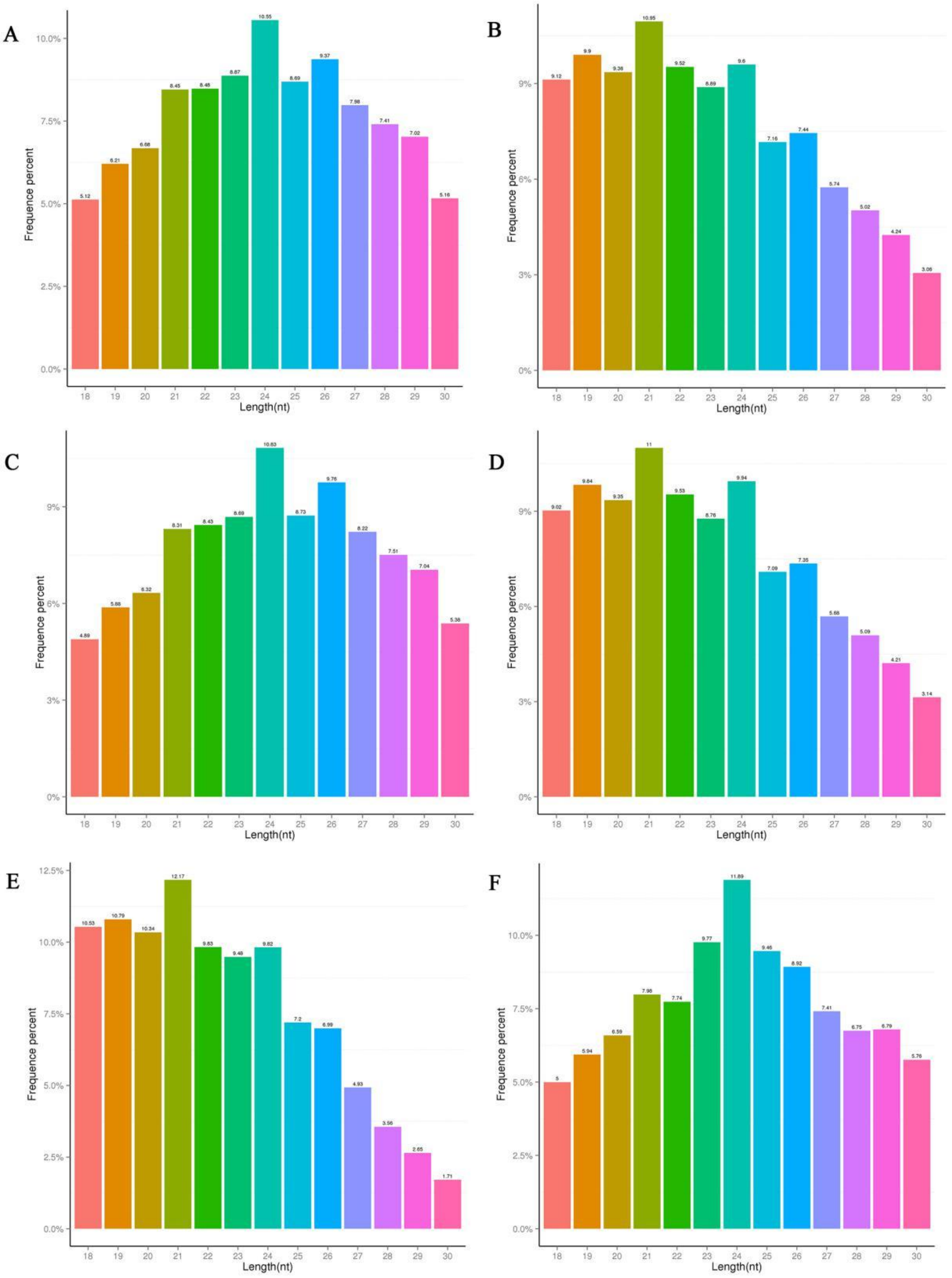

Figure S1. Length distribution of total sRNAs in constructed libraries. CK, LK6, LK12, LK24, LK48, and LK120 indicate control (0 h) and 6, 12, 24, 48, and 120 h of low-K treatment, respectively. (A, B, E, D, E, F) represent CK, LK6, LK12, LK24, LK48 and LK120, respectively.

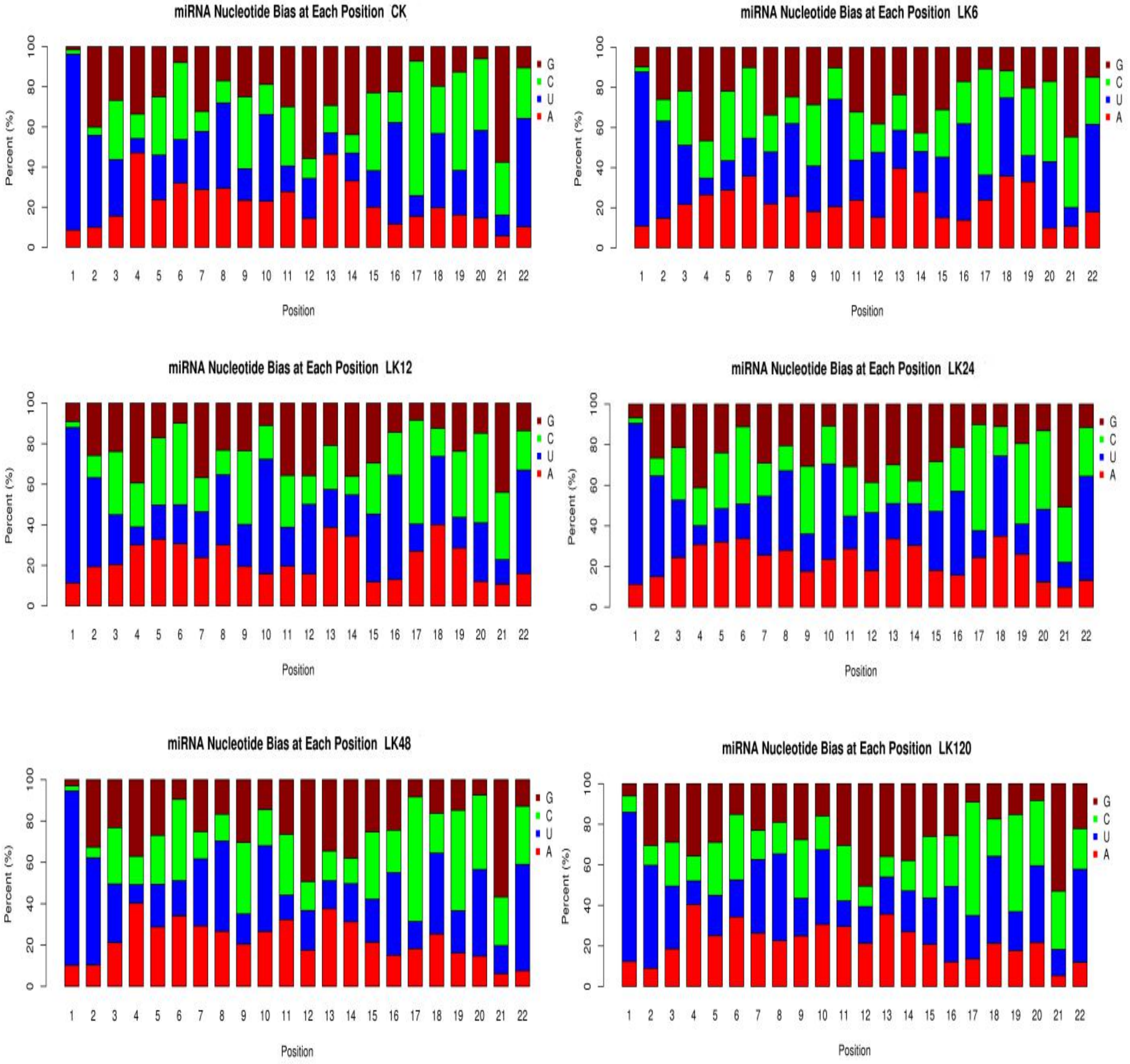

Figure S2. Base preference characterization for known miRNAs identified in this study. CK, LK6, LK12, LK24, LK48, and LK120 indicate control (0 h) and 6, 12, 24, 48 and 120 h of low-K treatment, respectively.

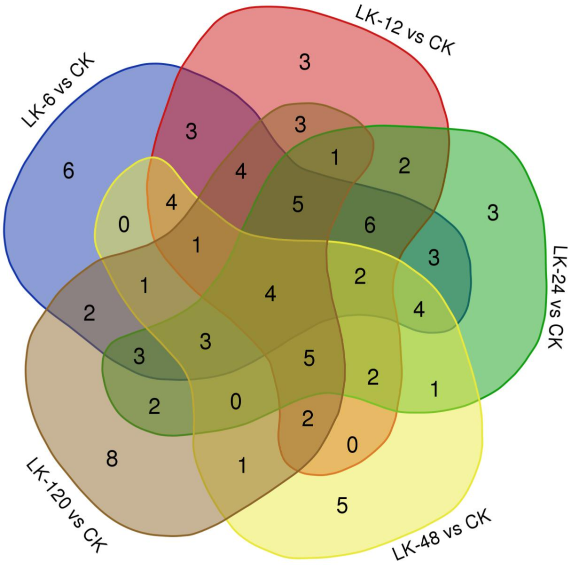

Figure S3. Venn diagram illustrating differential miRNA members identified in this study. CK, LK6, LK12, LK24, LK48, and LK120 indicate control (0 h) and 6, 12, 24, 48, and 120 h of low-K treatment, respectively. Data shown are averages from triplicate analyses.

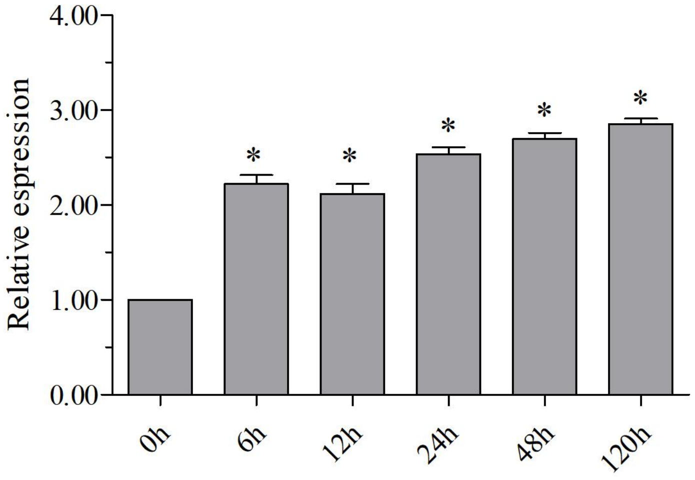

Figure S4. Transcript levels of *TaAKT1* in roots of wheat cultivar Kenong 9204 after 0, 6, 12, 24, 48, and 120 h of low-K treatment. Data are averages from triplicate analyses plus standard errors; * indicates significant difference at $P < 0.05$.

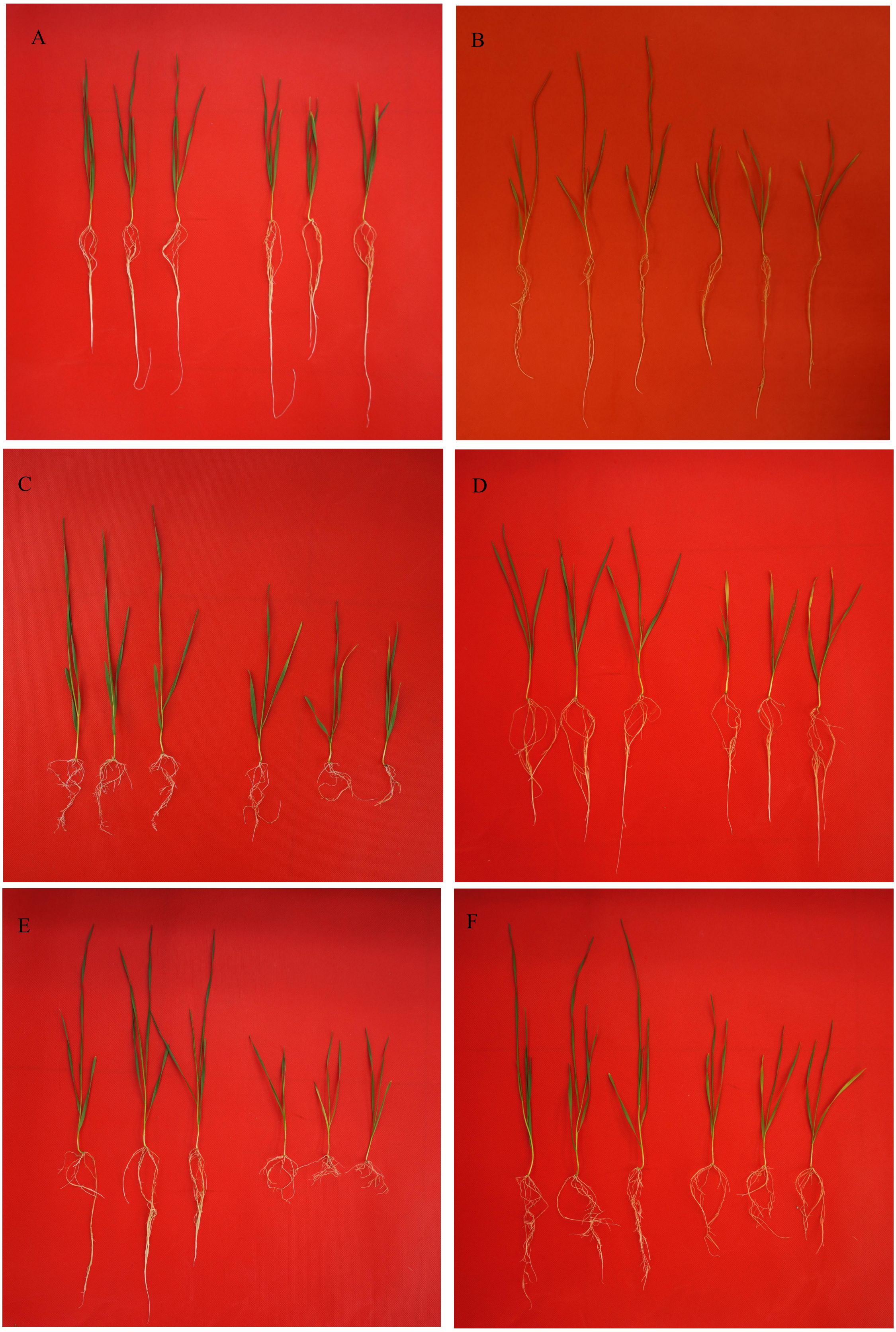

Figure S5. Phenotypes of seedlings of different wheat cultivars under control (MS) and low-potassium (LK) conditions. **(A)** Kenong 9204; **(B)** Aifeng 3; **(C)** Shannongda 5-6-5; **(D)** Jimai19; **(E)** Shannongjian 38-498; **(F)** Huaimai22.

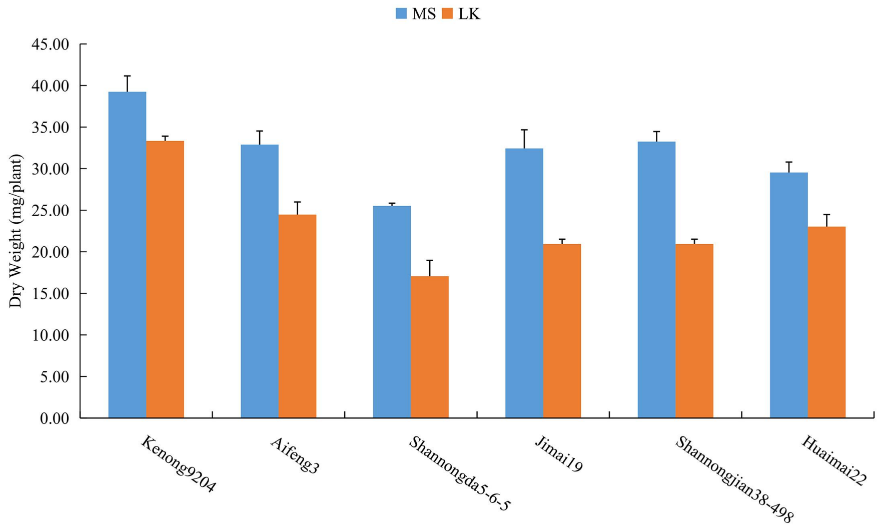

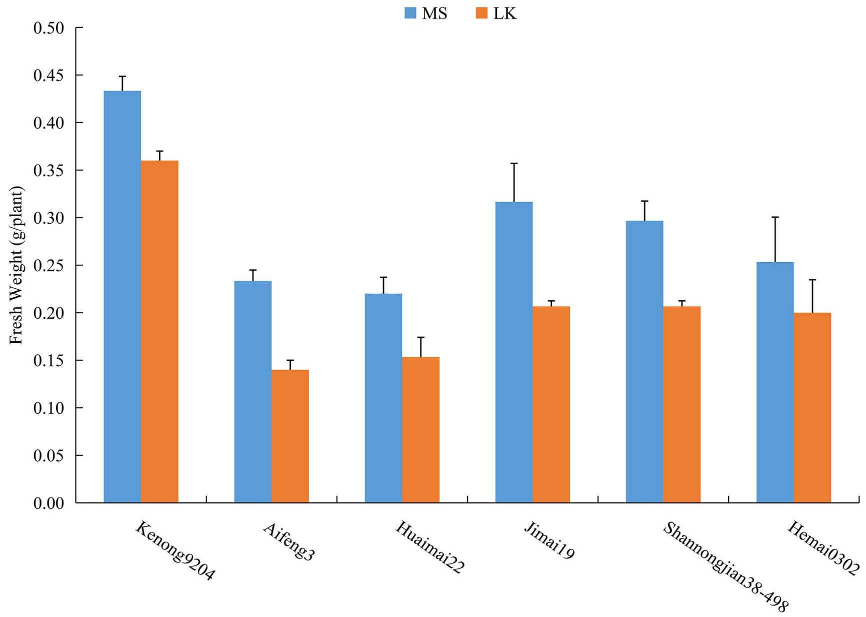

Figure S6. Fresh and dry weights of seedlings of various wheat cultivars under control (MS) and low-potassium (LK) conditions. **(A)** fresh weights; **(B)** dry weights. Data shown are averages from triplicate analyses.

Table S1. Sequence data output quality from six wheat (*Triticum aestivum* L.) libraries.

Sample	Reads	Bases	Error rate	Q20	Q30	GC content
CK	12334505	0.617G	0.01%	98.40%	96.43%	52.39%
LK-6	14373410	0.719G	0.01%	98.36%	96.30%	53.02%
LK-12	12443593	0.622G	0.01%	98.41%	96.41%	52.32%
LK-24	14464254	0.723G	0.01%	98.45%	96.51%	52.48%
LK-48	12822240	0.641G	0.01%	98.10%	95.71%	52.51%
LK-120	19340083	0.967G	0.01%	97.97%	95.27%	51.75%

Table 2. sRNA map information with reference to wheat (*Triticum aestivum* L.) cv. Chinese Spring genome.

Mapping to genome	CK	LK-6	LK-12	LK-24	LK-48	LK-120
Total reads	829998	6827553	8649698	9260600	7906183	7853652
Genome	6299908 (75.90%)	5824814 (85.31%)	6970293 (80.58%)	7394346 (79.85%)	6768763 (85.61%)	9998973 (81.56%)
known_miRNA	23867 (0.38%)	13661 (0.23%)	10968 (0.16%)	18103 (0.24%)	15329 (0.23%)	12971 (0.13%)
rRNA	956169 (15.18%)	1003458 (17.23%)	1082099 (15.52%)	1288030 (17.42%)	1180095 (17.43%)	2841579 (28.42%)
tRNA	9 (0.00%)	6 (0.00%)	7 (0.00%)	8 (0.00%)	10 (0.00%)	3 (0.00%)
snRNA	44842 (0.71%)	58422 (1.00%)	49332 (0.71%)	52248 (0.71%)	26595 (0.39%)	48547 (0.49%)
snoRNA	21647 (0.34%)	20968 (0.36%)	24807 (0.36%)	19461 (0.26%)	19122 (0.28%)	65451 (0.65%)
repeat	1158929 (18.40%)	959060 (16.47%)	1056067 (15.15%)	1194724 (16.16%)	1081329 (15.98%)	1287844 (12.88%)
NAT	2156889 (34.24%)	2364813 (40.60%)	2757132 (39.56%)	2719286 (36.78%)	2677737 (39.56%)	3089991 (30.90%)
novel_miRNA	6515 (0.10%)	2417 (0.04%)	2342 (0.03%)	3721 (0.05%)	3928 (0.06%)	3186 (0.03%)
TAS	18 (0.00%)	8 (0.00%)	6 (0.00%)	10 (0.00%)	12 (0.00%)	2 (0.00%)
exon	113987 (1.81%)	93987 (1.62%)	115136 (1.66%)	117098 (1.58%)	105431 (1.56%)	184595 (1.85%)
intron	275201 (4.36%)	301708 (5.18%)	566952 (8.14%)	432098 (5.85%)	323210 (4.78%)	417478 (4.18%)
other	1541835 (24.47%)	1006312 (17.28%)	1305445 (18.73%)	1549559 (20.96%)	1335965 (19.74%)	2047326 (20.48%)

Table S3. Numbers of mature and precursor miRNAs. **(A)** Numbers of known mature and precursor miRNAs; **(B)** Numbers of novel mature and precursor miRNAs.

(A)

TABLE S3A. Numbers of known mature and precursor miRNAs (Known)

Types	CK	LK-6	LK-12	LK-12	LK-48	LK-120
Mapped mature	66	67	62	73	71	65
Mapped hairpin	90	83	84	91	85	86

(B)

TABLE S3B. Numbers of mature and precursor miRNAs (Novel)

Types	CK	LK-6	LK-12	LK-12	LK-48	LK-120
Mapped mature	25	24	23	25	27	23
Mapped hairpin	26	29	25	29	28	26

Table S4. Differentially expressed miRNAs identified in this study. **(A)** Differentially expressed miRNAs in LK-6/CK comparison; (**B**) Differentially expressed miRNAs in LK-12/CK comparison; (**C**) Differentially expressed miRNAs in LK-24/CK comparison; **(D)** Differentially expressed miRNAs in LK-48/CK comparison; **(E)** Differentially expressed miRNAs in LK-120/CK comparison. CK, LK-6, LK-12, LK-24, LK-48, and LK-120 indicate control (0 h) and 6, 12, 24, 48, and 120 h of low-K treatment, respectively. LK-X/CK, comparison of miRNA expression between time point LK-X and CK.

(A)

Table S4A. Differentially expressed miRNAs identified in this study (LK-6/CK)

sRNA	LK-6	CK	log2.Fold_change.	p.value	q.value.
tae-miR9775	220.2528876	0	8.783	2.28E-54	3.03E-55
novel_30	165.1896657	0	8.368	1.82E-43	2.19E-44
tae-miR5049-3p	165.1896657	0	8.368	1.82E-43	2.19E-44
novel_50	110.1264438	0	7.783	1.35E-31	1.48E-32
tae-miR1122b-3p	55.06322191	0	6.783	3.63E-18	3.22E-19
tae-miR167b	55.06322191	0	6.783	3.63E-18	3.22E-19
tae-miR6197-5p	55.06322191	0	6.783	3.63E-18	3.22E-19
tae-miR9654a-3p	55.06322191	0	6.783	3.63E-18	3.22E-19
tae-miR9782	55.06322191	0	6.783	3.63E-18	3.22E-19
tae-miR9670-3p	3138.603649	333.9902141	3.2322	0	0
tae-miR531	1376.580548	250.4926605	2.4582	0	0
tae-miR1120c-5p	550.6322191	125.2463303	2.1363	1.00E-125	1.68E-126
tae-miR1133	495.5689972	125.2463303	1.9843	1.45E-107	2.31E-108
tae-miR9779	385.4425534	125.2463303	1.6217	1.84E-72	2.66E-73
tae-miR1130b-3p	715.8218849	250.4926605	1.5148	3.37E-126	5.80E-127
tae-miR396-5p	825.9483287	292.2414373	1.4989	3.86E-144	7.25E-145
tae-miR9772	8149.356843	2964.16315	1.4591	0	0
novel_33	110.1264438	41.74877676	1.3994	1.03E-19	9.84E-21
novel_13	4680.373863	1836.946177	1.3493	0	0
tae-miR1128	605.695441	250.4926605	1.2738	9.30E-94	1.45E-94
novel_18	991.1379944	417.4877676	1.2474	9.23E-150	1.78E-150
tae-miR9776	4405.057753	1878.694954	1.2294	0	0
tae-miR1120b-3p	275.3161096	125.2463303	1.1363	4.84E-40	5.62E-41
tae-miR1137a	2643.034652	1210.714526	1.1263	0	0
tae-miR171a	5616.448635	2588.424159	1.1176	0	0
tae-miR9773	1706.959879	834.9755351	1.0316	8.65E-222	1.97E-222
tae-miR159a	75436.61402	154470.474	-1.034	0	0
tae-miR7757-5p	11343.02371	25800.74404	-1.1856	1.90E-136	3.46E-137
novel_20	55.06322191	125.2463303	-1.1856	0.085899	0.0058939

sRNA	LK-6	CK	log2.Fold_change.	p.value	q.value.
novel_54	55.06322191	125.2463303	-1.1856	0.085899	0.0058939
tae-miR9653a-3p	1211.390882	3381.650917	-1.4811	9.38E-52	1.22E-52
tae-miR9778	50878.41705	149043.133	-1.5506	0	0
tae-miR9658-3p	1156.32766	3548.646024	-1.6177	3.65E-74	5.41E-75
tae-miR1127b-3p	55.06322191	208.7438838	-1.9226	8.17E-09	6.36E-10
novel_10	1541.770214	6387.562844	-2.0507	9.00E-266	2.21E-266
tae-miR9652-5p	110.1264438	500.9853211	-2.1856	5.18E-26	5.51E-27
novel_1	28357.55928	148542.1477	-2.3891	0	0
novel_14	28357.55928	148542.1477	-2.3891	0	0
tae-miR319	27751.86384	147373.182	-2.4088	0	0
tae-miR1121	110.1264438	626.2316514	-2.5075	4.38E-43	5.18E-44
tae-miR398	110.1264438	1294.212079	-3.5548	1.02E-156	2.04E-157
tae-miR408	220.2528876	7431.282263	-5.0764	0	0
novel_6	0	125.2463303	-7.9686	7.85E-24	7.83E-25
novel_21	0	41.74877676	-6.3837	2.60E-09	2.07E-10
novel_43	0	41.74877676	-6.3837	2.60E-09	2.07E-10
tae-miR1125	0	41.74877676	-6.3837	2.60E-09	2.07E-10
tae-miR1137b-5p	0	41.74877676	-6.3837	2.60E-09	2.07E-10
tae-miR1847-5p	0	41.74877676	-6.3837	2.60E-09	2.07E-10
tae-miR395b	0	41.74877676	-6.3837	2.60E-09	2.07E-10
novel_51	0	125.2463303	-7.9686	7.85E-24	7.83E-25
tae-miR9657a-3p	0	292.2414373	-9.191	4.76E-48	6.07E-49

(B)

Table S4B. Differentially expressed miRNAs identified in this study (LK-12/CK)

sRNA	LK-12	CK	log2.Fold_change.	p.value	q.value
tae-miR395a	209.1334159	0	8.7083	1.04E-49	2.33E-49
novel_50	139.4222773	0	8.1233	3.72E-36	7.21E-36
tae-miR1122b-3p	139.4222773	0	8.1233	3.72E-36	7.21E-36
tae-miR1123	69.71113864	0	7.1233	8.57E-21	1.32E-20
tae-miR9654a-3p	69.71113864	0	7.1233	8.57E-21	1.32E-20
tae-miR9670-3p	2858.156684	314.5881761	3.1835	0	0
tae-miR395b	209.1334159	39.32352201	2.411	5.68E-47	1.23E-46
tae-miR9674a-5p	209.1334159	39.32352201	2.411	5.68E-47	1.23E-46
novel_54	557.6891092	117.970566	2.241	1.20E-115	3.59E-115
tae-miR531	1045.66708	235.9411321	2.1479	4.30E-208	1.53E-207

sRNA	LK-12	CK	log2.Fold_change.	p.value	q.value
tae-miR9779	487.9779705	117.970566	2.0484	1.55E-94	4.25E-94
tae-miR1137a	4322.090596	1140.382138	1.9222	0	0
tae-miR1120b-3p	418.2668319	117.970566	1.826	4.87E-74	1.26E-73
novel_43	139.4222773	39.32352201	1.826	7.86E-26	1.35E-25
novel_49	139.4222773	39.32352201	1.826	7.86E-26	1.35E-25
tae-miR1137b-5p	139.4222773	39.32352201	1.826	7.86E-26	1.35E-25
tae-miR1120c-5p	348.5556932	117.970566	1.563	2.05E-54	4.69E-54
novel_34	3067.2901	1101.058616	1.4781	0	0
tae-miR9774	1812.489605	668.4998742	1.439	7.72E-256	2.85E-255
tae-miR9773	2091.334159	786.4704402	1.411	7.57E-290	2.91E-289
tae-miR9660-5p	278.8445546	117.970566	1.241	4.52E-36	8.51E-36
tae-miR9772	6413.424755	2791.970063	1.1998	0	0
tae-miR9676-5p	1324.511634	589.8528301	1.167	6.71E-156	2.15E-155
novel_19	348.5556932	157.294088	1.1479	8.90E-42	1.78E-41
tae-miR396-5p	557.6891092	275.2646541	1.0186	2.29E-59	5.36E-59
novel_17	348.5556932	707.8233962	-1.022	1.95E-05	2.28E-05
tae-miR9653a-3p	1324.511634	3185.205283	-1.2659	1.20E-45	2.50E-45
tae-miR9778	57232.84483	140384.9736	-1.2945	0	0
tae-miR159a	46148.77378	145497.0314	-1.6566	0	0
novel_1	38619.97081	139913.0913	-1.8571	0	0
novel_14	38619.97081	139913.0913	-1.8571	0	0
tae-miR319	36249.79209	138812.0327	-1.9371	0	0
tae-miR9678-3p	69.71113864	275.2646541	-1.9814	3.95E-15	5.66E-15
novel_10	1394.222773	6016.498868	-2.1095	0	0
tae-miR398	278.8445546	1219.029182	-2.1282	1.65E-71	4.18E-71
tae-miR408	418.2668319	6999.586918	-4.0648	0	0
novel_6	0	117.970566	-7.8823	2.23E-23	3.57E-23
novel_33	0	39.32352201	-6.2973	3.34E-09	4.25E-09
tae-miR1125	0	39.32352201	-6.2973	3.34E-09	4.25E-09
tae-miR1127a	0	39.32352201	-6.2973	3.34E-09	4.25E-09
tae-miR1847-5p	0	39.32352201	-6.2973	3.34E-09	4.25E-09
novel_15	0	78.64704402	-7.2973	1.28E-16	1.89E-16
tae-miR1122a	0	78.64704402	-7.2973	1.28E-16	1.89E-16
tae-miR397-5p	0	78.64704402	-7.2973	1.28E-16	1.89E-16
novel_26	0	117.970566	-7.8823	2.23E-23	3.57E-23
tae-miR9679-5p	0	117.970566	-7.8823	2.23E-23	3.57E-23
tae-miR1122c-3p	0	157.294088	-8.2973	1.07E-29	1.93E-29

(C)

Table S4C. Differentially expressed miRNAs identified in this study (LK-24/CK)

sRNA	LK-24	CK	log2.Fold_change.	p.value	q.value
novel_50	157.2264743	0	8.2967	8.44E-41	2.02E-40
tae-miR5384-3p	117.9198557	0	7.8817	1.61E-32	3.45E-32
tae-miR9775	117.9198557	0	7.8817	1.61E-32	3.45E-32
tae-miR9782	117.9198557	0	7.8817	1.61E-32	3.45E-32
tae-miR395a	78.61323715	0	7.2967	1.52E-23	2.58E-23
tae-miR5049-3p	78.61323715	0	7.2967	1.52E-23	2.58E-23
tae-miR1118	78.61323715	0	7.2967	1.52E-23	2.58E-23
tae-miR9657c-3p	78.61323715	0	7.2967	1.52E-23	2.58E-23
novel_30	39.30661858	0	6.2967	1.82E-13	2.52E-13
tae-miR167b	39.30661858	0	6.2967	1.82E-13	2.52E-13
tae-miR6197-5p	39.30661858	0	6.2967	1.82E-13	2.52E-13
tae-miR9666b-3p	39.30661858	0	6.2967	1.82E-13	2.52E-13
tae-miR1120b-3p	707.5191344	121.7014927	2.5394	1.09E-170	3.86E-170
tae-miR9674a-5p	196.5330929	40.56716424	2.2764	2.72E-45	7.00E-45
tae-miR9670-3p	1257.811794	324.5373139	1.9545	1.62E-249	6.93E-249
tae-miR1120c-5p	432.3728043	121.7014927	1.8289	1.06E-82	3.13E-82
tae-miR9774	2279.783877	689.6417921	1.725	0	0
tae-miR9772	8568.84285	2880.268661	1.5729	0	0
tae-miR171a	6957.271488	2515.164183	1.4679	0	0
tae-miR1137a	2947.996393	1176.447763	1.3253	0	0
novel_19	393.0661858	162.268657	1.2764	1.60E-56	4.22E-56
novel_34	2672.850063	1135.880599	1.2346	0	0
tae-miR531	550.2926601	243.4029854	1.1769	2.08E-73	5.96E-73
tae-miR9773	1808.104455	811.3432848	1.1561	1.88E-233	7.46E-233
novel_13	3773.435383	1784.955227	1.08	0	0
tae-miR9675-3p	8293.69652	3935.014931	1.0756	0	0
tae-miR9676-5p	1257.811794	608.5074636	1.0476	1.98E-150	6.59E-150
tae-miR9657a-3p	117.9198557	283.9701497	-1.2679	0.00025969	0.00029393
tae-miR9658-3p	1218.505176	3448.20896	-1.5007	1.74E-68	4.85E-68
novel_8	393.0661858	1135.880599	-1.531	3.49E-25	6.53E-25
novel_26	39.30661858	121.7014927	-1.6305	0.00017921	0.00021096
tae-miR9679-5p	39.30661858	121.7014927	-1.6305	0.00017921	0.00021096
tae-miR9778	43119.36058	144824.7763	-1.7479	0	0
novel_10	1768.797836	6206.776129	-1.8111	3.99E-213	1.52E-212
novel_1	40682.35023	144337.9704	-1.827	0	0

sRNA	LK-24	CK	log2.Fold_change.	p.value	q.value
novel_14	40643.04361	144337.9704	-1.8284	0	0
novel_17	157.2264743	730.2089563	-2.2155	1.87E-42	4.59E-42
tae-miR1127b-3p	39.30661858	202.8358212	-2.3675	1.13E-14	1.66E-14
tae-miR319	27593.24624	143202.0898	-2.3757	0	0
tae-miR398	196.5330929	1257.582091	-2.6778	3.48E-104	1.12E-103
tae-miR408	157.2264743	7220.955234	-5.5213	0	0
novel_6	0	121.7014927	-7.9272	1.33E-23	2.39E-23
novel_21	0	40.56716424	-6.3422	2.95E-09	3.71E-09
tae-miR1122a	0	81.13432848	-7.3422	9.29E-17	1.41E-16
novel_51	0	121.7014927	-7.9272	1.33E-23	2.39E-23
tae-miR9779	0	121.7014927	-7.9272	1.33E-23	2.39E-23

(D)

Table S4D. Differentially expressed miRNAs identified in this study (LK-48/CK)

sRNA	LK-48	CK	log2.Fold_change.	p.value	q.value
novel_50	184.9342804	0	8.5309	1.36E-41	5.07E-42
tae-miR9652-3p	138.7007103	0	8.1158	4.32E-33	1.43E-33
tae-miR395a	92.4671402	0	7.5309	6.87E-24	2.05E-24
tae-miR9782	92.4671402	0	7.5309	6.87E-24	2.05E-24
novel_30	46.2335701	0	6.5309	1.40E-13	2.97E-14
tae-miR1118	46.2335701	0	6.5309	1.40E-13	2.97E-14
tae-miR1131	46.2335701	0	6.5309	1.40E-13	2.97E-14
tae-miR6197-5p	46.2335701	0	6.5309	1.40E-13	2.97E-14
tae-miR9659-3p	46.2335701	0	6.5309	1.40E-13	2.97E-14
tae-miR9775	46.2335701	0	6.5309	1.40E-13	2.97E-14
novel_33	138.7007103	35.36142168	1.9717	2.81E-21	7.99E-22
tae-miR9774	1988.043514	601.1441686	1.7256	5.39E-245	5.36E-245
novel_51	323.6349907	106.0842651	1.6092	1.66E-38	6.00E-39
tae-miR531	601.0364113	212.1685301	1.5022	5.57E-65	2.77E-65
novel_19	369.8685608	141.4456867	1.3868	2.61E-37	9.15E-38
tae-miR1127a	92.4671402	35.36142168	1.3868	1.75E-10	3.43E-11
tae-miR1137b-5p	92.4671402	35.36142168	1.3868	1.75E-10	3.43E-11
tae-miR9674a-5p	92.4671402	35.36142168	1.3868	1.75E-10	3.43E-11
tae-miR1130b-3p	508.5692711	212.1685301	1.2612	1.25E-45	4.99E-46
tae-miR9772	5316.860562	2510.66094	1.0825	0	0
tae-miR319	61629.34895	124825.8185	-1.0182	0	0
tae-miR159a	61629.34895	124825.8185	-1.0182	0	0

sRNA	LK-48	CK	log2.Fold_change.	p.value	q.value
novel_26	46.2335701	106.0842651	-1.1982	0.00034971	5.28E-05
novel_54	46.2335701	106.0842651	-1.1982	0.00034971	5.28E-05
tae-miR1120a	46.2335701	106.0842651	-1.1982	0.00034971	5.28E-05
tae-miR1120b-3p	46.2335701	106.0842651	-1.1982	0.00034971	5.28E-05
tae-miR9660-5p	46.2335701	106.0842651	-1.1982	0.00034971	5.28E-05
tae-miR9679-5p	46.2335701	106.0842651	-1.1982	0.00034971	5.28E-05
novel_6	46.2335701	106.0842651	-1.1982	0.00034971	5.28E-05
novel_17	46.2335701	106.0842651	-1.1982	0.00034971	5.28E-05
tae-miR9657a-3p	92.4671402	247.5299518	-1.4206	4.10E-11	8.43E-12
tae-miR1127b-3p	46.2335701	176.8071084	-1.9352	5.99E-14	1.46E-14
tae-miR1847-5p	0	35.36142168	-6.1441	4.54E-09	8.60E-10
tae-miR171b	0	70.72284337	-7.1441	3.04E-16	7.73E-17
tae-miR9777	0	70.72284337	-7.1441	3.04E-16	7.73E-17

(E)

Table S4E. Differentially expressed miRNAs identified in this study (LK-120/CK)

sRNA	LK-120	CK	log2.Fold_change.	p.value	q.value
tae-miR395a	225.6771967	0	8.8181	1.09E-50	2.36E-51
tae-miR5049-3p	169.2578975	0	8.4031	1.93E-40	3.60E-41
tae-miR9652-3p	112.8385984	0	7.8181	2.63E-29	4.29E-30
tae-miR1117	56.41929918	0	6.8181	9.34E-17	1.20E-17
tae-miR5084	56.41929918	0	6.8181	9.34E-17	1.20E-17
tae-miR1138	56.41929918	0	6.8181	9.34E-17	1.20E-17
tae-miR9666b-3p	56.41929918	0	6.8181	9.34E-17	1.20E-17
tae-miR9673-5p	56.41929918	0	6.8181	9.34E-17	1.20E-17
tae-miR531	2087.51407	224.2272295	3.2188	0	0
tae-miR9674a-5p	338.5157951	37.37120492	3.1792	3.38E-82	8.26E-83
tae-miR9774	3949.350943	635.3104836	2.6361	0	0
tae-miR395b	225.6771967	37.37120492	2.5943	3.46E-48	7.30E-49
tae-miR9773	2764.54566	747.4240983	1.887	0	0
tae-miR396-5p	959.1280861	261.5984344	1.8744	2.40E-150	6.69E-151
novel_13	5246.994824	1644.333016	1.674	0	0
novel_34	3272.319353	1046.393738	1.6449	0	0
novel_51	338.5157951	112.1136147	1.5943	1.00E-46	2.01E-47
novel_43	112.8385984	37.37120492	1.5943	1.16E-16	1.44E-17
novel_49	112.8385984	37.37120492	1.5943	1.16E-16	1.44E-17

sRNA	LK-120	CK	log2.Fold_change.	p.value	q.value
novel_8	2651.707062	1046.393738	1.3415	3.47E-298	1.35E-298
tae-miR1121	1410.48248	560.5680737	1.3312	2.90E-158	8.40E-159
tae-miR9779	282.0964959	112.1136147	1.3312	4.37E-33	7.43E-34
tae-miR9772	6601.058004	2653.355549	1.3149	0	0
tae-miR9653b	3723.673746	1569.590606	1.2463	0	0
tae-miR1137a	2538.868463	1083.764943	1.2281	1.46E-261	5.20E-262
tae-miR1120b-3p	225.6771967	112.1136147	1.0093	1.08E-20	1.53E-21
tae-miR9778	64092.32387	133415.2015	-1.0577	0	0
tae-miR319	60650.74662	131920.3534	-1.1211	0	0
tae-miR9672b	21608.59159	56056.80737	-1.3753	0	0
tae-miR9652-5p	169.2578975	448.454459	-1.4057	5.75E-14	6.92E-15
tae-miR9670-3p	112.8385984	298.9696393	-1.4057	8.54E-10	9.68E-11
tae-miR9669-5p	1297.643881	3886.605311	-1.5826	1.03E-144	2.79E-145
tae-miR9672a-3p	225.6771967	710.0528934	-1.6537	1.22E-30	2.04E-31
tae-miR9678-3p	56.41929918	261.5984344	-2.2131	6.47E-21	9.37E-22
novel_21	0	37.37120492	-6.2239	3.96E-09	4.24E-10
tae-miR1125	0	37.37120492	-6.2239	3.96E-09	4.24E-10
tae-miR1127a	0	37.37120492	-6.2239	3.96E-09	4.24E-10
tae-miR1847-5p	0	37.37120492	-6.2239	3.96E-09	4.24E-10
novel_26	0	112.1136147	-7.8088	4.72E-23	7.10E-24
tae-miR9660-5p	0	112.1136147	-7.8088	4.72E-23	7.10E-24
tae-miR9679-5p	0	112.1136147	-7.8088	4.72E-23	7.10E-24
tae-miR1122c-3p	0	149.4848197	-8.2239	3.00E-29	4.79E-30
tae-miR1127b-3p	0	186.8560246	-8.5458	4.03E-35	7.16E-36
tae-miR1130b-3p	0	224.2272295	-8.8088	9.76E-41	1.86E-41
tae-miR9657a-3p	0	261.5984344	-9.0312	3.84E-46	7.51E-47

Table S5. Wheat (*Triticum aestivum* L.) miRNAs differentially expressed at three or more time points under low-K conditions. (A) Up-regulated miRNAs; (B) down-regulated miRNAs.

(A)

Table S5A. Wheat (*Triticum aestivum* L.) miRNAs differentially expressed at three or more time points under low-K conditions (up-regulated)

miRNA	log2.Fold_change (LK-6/CK)	log2.Fold_change (LK-12/CK)	log2.Fold_change (LK-24/CK)	log2.Fold_change (LK-48/CK)	log2.Fold_change (LK-120/CK)
novel_50	7.78	8.12	8.3	8.53	--
miRNA531	2.46	2.15	1.18	1.5	3.22
miRNA9772	1.46	1.2	1.57	1.08	1.31
miRNA9670-3p	3.23	3.18	1.95	--	1.41
miRNA9773	1.03	1.41	1.16	--	1.89

(B)

Table S5B. Wheat (*Triticum aestivum* L.) miRNAs differentially expressed at three or more time points under low-K conditions (down-regulated)

miRNA	log2.Fold_change (LK-6/CK)	log2.Fold_change (LK-12/CK)	log2.Fold_change (LK-24/CK)	log2.Fold_change (LK-48/CK)	log2.Fold_change (LK-120/CK)
novel_17	--	-1.02	-2.22	-1.19	--
miRNA408	-5.08	-4.068	-5.52	--	--
miRNA1127a	--	-6.3	--	-1.39	-6.22
miRNA159a	-1.03	-1.66	--	-1.02	--
miRNA319	-2.41	-1.94	-2.38	-1.02	-1.12
miRNA398	-3.55	-2.13	-2.68	--	--
miRNA9778	-1.55	-1.29	-1.75	--	-1.06

Table S6. Target genes of differentially expressed miRNAs under low-K conditions.

TABLE S6 | Target genes of differentially expressed miRNAs under low-K conditions.

MiRNA	Seq	target genes	description
novel_17	UGUGCCUGGCUCCCUGUAUGCC	TRIAE_CS42_3B_TGACv1_224001_AA0790760	RING-type E3 ubiquitin transferase
		TRIAE_CS42_6DS_TGACv1_543780_AA1744110	Glycosyltransferase
novel_50	GCGCGCCUGUCGGGACCC	unknown	unknown
miRNA319	UUGGACUGAAGGGAGCUCCCU	TRIAE_CS42_3B_TGACv1_224322_AA0795230	Auxin-responsive protein
		TRIAE_CS42_7AL_TGACv1_558123_AA1790390	Peroxidase
miRNA531	CGCUCGCCGGAGCAGCGUGCA	TRIAE_CS42_3AL_TGACv1_195693_AA0652800	Alpha-mannosidase I
		TRIAE_CS42_3B_TGACv1_220852_AA0721380	Metal-nicotine transporter
miRNA9773	UUUGUUUUUAUGUUAUUUUGUGAA	TRIAE_CS42_7AL_TGACv1_556846_AA1772010	Transposons TNT 1-94
		TRIAE_CS42_4DL_TGACv1_342809_AA1122550	Formalin 6
miRNA9670-3p	AGGUGGAAUACUUGAAGAAGA	TRIAE_CS42_4BS_TGACv1_327960_AA1079810	Serine/threonine protein kinase
miRNA398	UGUGUUCUCAGGUCGCCCCCG	TRIAE_CS42_2DS_TGACv1_179233_AA0605480	Superoxide dismutase [Cu-Zn]
		TRIAE_CS42_3B_TGACv1_224783_AA0801500	Chloride channel protein
miRNA159a	UUUGGAUUGAAGGGAGCUCUG	TRIAE_CS42_2AS_TGACv1_112965_AA0348840	RNA-dependent RNA polymerase
		TRIAE_CS42_7BL_TGACv1_579247_AA1905780	Peroxidase
miRNA9778	UGCAUCAUCUCGAACUCGUCG	TRIAE_CS42_7AS_TGACv1_570511_AA1836830	Anti-pathogenic protein
		TRIAE_CS42_7DL_TGACv1_603394_AA1982670	Anti-pathogenic protein
miRNA408	CUGCACUGCCUCUUCCCUGGC	TRIAE_CS42_U_TGACv1_641280_AA2090580	Protein phosphatase
		TRIAE_CS42_6BL_TGACv1_501598_AA1619180	NAC domain protein
miRNA9776	UUGGACGAGGAUGUGCAACUG	TRIAE_CS42_4BS_TGACv1_328595_AA1090730	Lipoxygenase
		TRIAE_CS42_1DL_TGACv1_064144_AA0232890	Auxin-responsive protein
miRNA1133	CAUAUACUCCCUCCGUCCGAAA	TRIAE_CS42_3B_TGACv1_221753_AA0749220	Protein DETOXIFICATION
		TRIAE_CS42_4BS_TGACv1_330238_AA1106670	Serine/threonine-protein kinase

Table S7. List of PCR primers used in this study.

Table S7. List of PCR primers used in this study

Name	F(5'-3')	R(5'-3')
TeamiR319	UUCACUCGAGCACACAGUAGA	GCTCATTCGCACAGTACATAT
TeamiR1133	TTCTTAGTGATAGTGGTCAAT	TAGTGCTAGATACATCCATT
TeamiR9776	TAGCTAGAGCTTGGACGAGGA	AGGTAGAGCTTGGGCATGGA
novel_17	TGTGCCTGGCTCCCTGTATGCC	CATGCTTGGCCCCTTGCACGC
TeamiR397-5p	CGCAAAGGTGCCATTGAGTGCA	GCAGAGGCGTCATTGTGTGCAG
TeamiR398	AGACGCGAGGAAATTCCTGCG	GCGAGGAGGCTCCAGCGGG
TeamiR159a	GTGGAGCTCCTATCATTCCAAT	ATGCAGAGCTCCCTTCAATCCAA
TeamiR408	ATTTTGTGAGTGGAGAGGGG	AGAGAGGGGGAGGGAGAGATT
NtKAT3	TGGTGGAGTTATCCTCTGCATT	CTAACCACACAAATTCCATAAGC
NtKAT5	GTGTCCCGCACTCGCATTTAAT	TACTATAAAGGCAAGGGGTAAC
NtKAT7	CCTCCATAAGAAAGATGAGGAGT	ATGAGGTACGTTGAAGATCACAC
NtKAT11	CGGAGGCTTTGGTTATGTGGA	GGACCAGATGAGAAGGGAACA
TaKAT 1	GGTCTACTCTGCGTGGGTCT	ATTTCTGAGGGATGGTGG
TRIAE_CS42_6DS_TGACv1_543780_AA1744110 (*GT*, encoding glycosyltransferase, target of novel_17)	CAAGACTTCTCAACCGAAGCA	TGAGCTCCAAGACTCGCAAC
TRIAE_CS42_7AL_TGACv1_558123_AA1790390 (*POD*, encoding peroxidase, target of miR319)	ATACGCAAATGTGGCATCGTC	ATTCCACGTCAGAGAACAAGCA
TRIAE_CS42_7AL_TGACv1_556774_AA1770580 (*HAK*, encoding potassium transporter, target of miR397-5p)	ACAAAACACTTGGGCAATGCATC	CCGATTGCCAGGAATATGCTCT
TRIAE_CS42_2DS_TGACv1_179233_AA0605480 (*SOD*, encoding superoxide dismutase [Cu-Zn], target of miR398)	TTATTTCGTCCCCGTGCAG	CCTCCAACTTAAACGACGCAAA
TRIAE_CS42_2AS_TGACv1_112965_AA0348840 (*RdRp*, encoding RNA-dependent RNA polymerase, target of miR159a)	TCCAGAATAGCATGAGCCGAA	CAACGAACATTCTAGCACGAG
TRIAE_CS42_U_TGACv1_641280_AA2090580 (*PP*, encoding protein phosphatase, target of miR408)	ACATGCTCCGACTGTCACC	CGTACCTATCCTTCCGGTCCC
TRIAE_CS42_1DL_TGACv1_064144_AA0232890 (*ARP*, encoding auxin-responsive protein, target of miR9776)	GCAGACCGCAGTTCATCACA	ACAAGATATGCAGCGTGTCCT
TRIAE_CS42_4BS_TGACv1_330238_AA1106670 (*STPK*, encoding serine/threonine-protein kinase, target of 1133)	CAGCCTTCCCATCACCGAGA	ACGATCATCTTCACCACGGTT
GAPDH	TAAGGGTGGTGCCAAGAAGGT	AGCAAGAGGAGCAAGGCAGT
